# Crystal structure of a host–guest complex between mephedrone hydro­chloride and a tetra­phospho­nate cavitand

**DOI:** 10.1107/S2056989019001464

**Published:** 2019-01-29

**Authors:** Elisa Biavardi, Chiara Massera

**Affiliations:** a Dipartimento di Scienze Chimiche, della Vita e della Sostenibilità Ambientale, Università di Parma, Parco Area delle Scienze 17/A, 43124 Parma, Italy

**Keywords:** crystal structure, tetra­phospho­nate cavitands, inclusion compounds, mephedrone, illicit drugs

## Abstract

A new supra­molecular complex (I) between the tetra­phospho­nate cavitand Tiiii[C_3_H_7_,CH_3_,C_6_H_5_] [systematic name: 2,8,14,20-tetra­propyl-5,11,17,23-tetra­methyl-6,10:12,16:18,22:24,4-tetra­kis­(phenyl­phospho­nato-*O*,*O*′)resorcin[4]arene] and mephedrone hydro­choride {C_11_H_16_NO^+^·Cl^−^; systematic name: meth­yl[1-(4-methyl­phen­yl)-1-oxopropan-2-yl]aza­nium chloride} has been obtained and characterized both in solution and in the solid state. The complex of general formula (C_11_H_16_NO)@Tiiii[C_3_H_7_,CH_3_,C_6_H_5_]Cl·CH_3_OH or C_11_H_16_NO^+^·Cl^−^·C_68_H_68_O_12_P_4_·CH_3_OH, crystallizes in the monoclinic space group *P*2_1_/*c* with one lattice methanol mol­ecule per cavitand, disordered over two positions with occupancy factors of 0.665 (6) and 0.335 (6). The mephedrone guest inter­acts with the P=O groups at the upper rim of the cavitand through two charge-assisted N—H⋯O hydrogen bonds, while the methyl group directly bound to the amino moiety is stabilized inside the π basic cavity *via* cation⋯π inter­actions. The chloride counter-anion is located between the alkyl legs of the cavitand, forming C—H⋯Cl inter­actions with the aromatic and methyl­enic H atoms of the lower rim. The chloride anion is also responsible for the formation of a supra­molecular chain along the *b*-axis direction through C—H⋯Cl inter­actions involving the phenyl substituent of one phospho­nate group. C—H⋯O and C—H⋯π inter­actions between the guest and adjacent cavitands contribute to the formation of the crystal structure.

## Abstract 2

The mol­ecular recognition properties of the tetra­phospho­nate cavitand Tiiii[C_3_H_7_,CH_3_,C_6_H_5_] towards mephedrone hydro­chloride, an illicit drug belonging to the amphetamine family, have been analysed in the solid state through the detailed analysis of the crystal and mol­ecular structure of the resulting supra­molecular compound, and in solution *via* NMR studies.

## Chemical context   

Mephedrone (2-methyl­amino-1-*p*-tolyl­propan-1-one), often abbreviated as 4-MMC, the acronym of 4-methyl methcathinone, is a synthetic drug belonging to the family of methamphetamines known for its stimulant effects (Winstock *et al.*, 2010 ▸; Morris, 2010 ▸; Wood *et al.*, 2010 ▸). It can be considered a ‘designer drug’, that is, a compound resulting from the chemical modification of an existing drug, which in this case is cathinone, a natural alkaloid found in the plant *Catha edulis.* As a result of the major impact these substances have on human health and social security, it is extremely important to have sensitive, selective and fast methods to identify them as a class, independently from all the synthetic modifications that can be devised to market them and to bypass the legal restrictions to which the parent compounds are subjected. Among the existing analytical methods used to detect 4-MMC in human biological samples or in different media (water, mixtures of powders, *etc*), solid-phase extraction (SPE) and liquid chromatography combined with mass spectrometry (LC/MS) are the most common, as can be seen from the extended literature which has been published on the subject in the past few years (Kolmonen *et al.*, 2009 ▸; Singh *et al.*, 2010 ▸; Santali *et al.*, 2011 ▸; Frison *et al.*, 2011 ▸; Strano-Rossi *et al.*, 2012 ▸; Power *et al.*, 2012 ▸; Perera *et al.*, 2012 ▸; Lua *et al.*, 2012 ▸; Vircks & Mulligan, 2012 ▸; Concheiro *et al.*, 2013 ▸; Mayer *et al.*, 2013 ▸; Mwenesongole *et al.*, 2013 ▸; Pedersen *et al.*, 2013 ▸; Kanu *et al.*, 2013 ▸; Strano-Rossi *et al.*, 2014 ▸; de Castro *et al.*, 2014 ▸; Mercolini *et al.*, 2016 ▸; Salomone *et al.*, 2016 ▸; Fontanals *et al.*, 2017 ▸; Lendoiro *et al.*, 2017 ▸; Mercieca *et al.*, 2018 ▸; Robin *et al.*, 2018 ▸). Recently, the group of Professor Dalcanale has reported a new method to detect methamphetamine salts with extremely high selectivity in water, using cavitand-grafted silicon micro­canti­levers (Biavardi *et al.*, 2014 ▸); more precisely, MDMA (methyl­enedi­oxy­methamphetamine), cocaine, amphetamine, and 3-fluoro­methamphetamine hydro­chlorides have been successfully detected in this way. This method takes advantage of the ability shown by tetra­phospho­nate cavitands to selectively recognize the ^+^NH_2_—CH_3_ group (^+^NH*R*—CH_3_ in the case of cocaine) common to all the above-mentioned drug salts through the concomitant formation of CH_3_⋯π inter­actions and hydrogen bonding. Indeed, resorcinarene-based cavitands (Cram, 1983 ▸; Cram & Cram, 1994 ▸) decorated at the upper rim with phospho­nate groups or quinoxaline moieties have long been exploited for their mol­ecular recognition properties towards charged and neutral mol­ecules (Dutasta, 2004 ▸; Vachon *et al.*, 2011 ▸; Melegari *et al.*, 2013 ▸; Pinalli *et al.*, 2016 ▸; Tudisco *et al.*, 2016 ▸; Trzciński *et al.*, 2017 ▸; Pinalli *et al.*, 2018 ▸; Wu *et al.*, 2012 ▸; Clément *et al.*, 2015 ▸). In order to further assess the recognition properties of tetra­phospho­nate cavitands towards quaternary ammonium salts of social inter­est, the supra­molecular complex between Tiiii[C_3_H_7_, CH_3_, C_6_H_5_] and mephedrone hydro­chloride is herein reported and analysed, both in the solid state through the detailed analysis of its crystal and mol­ecular structure, and in solution *via* NMR studies.
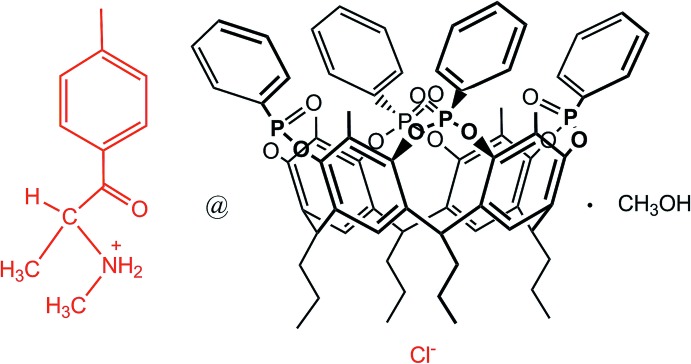


## Structural commentary   

**Figure 1:**
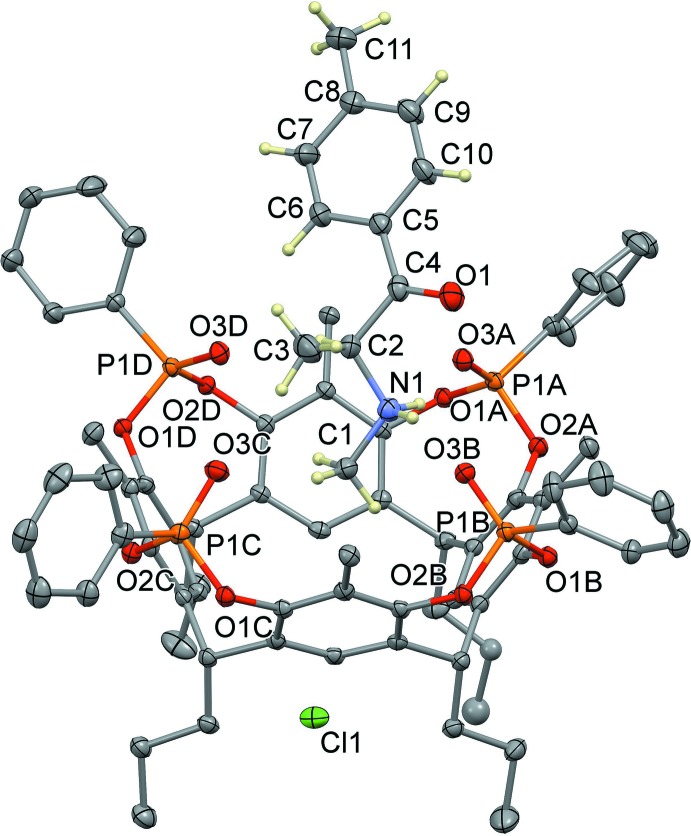
*ORTEP* view of (C_11_H_16_NO)@Tiiii[C_3_H_7_,CH_3_,*C*_6_H_5_]Cl (I) with partial atom-labelling scheme and anisotropic displacement parameters drawn at the 20% probability level. The solvent mol­ecules and the H atoms of the cavitand are omitted for clarity; only one orientation of the disordered alkyl chain is shown.

**Figure 2:**
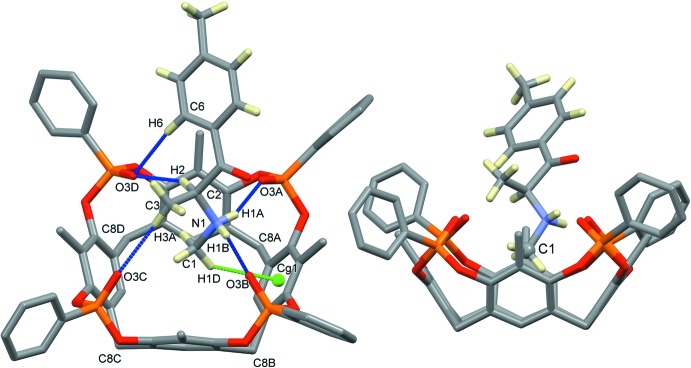
Left: view of the main host–guest supra­molecular inter­actions shown as blue and green dotted lines. Only relevant H atoms are shown, while the alkyl chains, the chloride anion and the methanol lattice mol­ecules have been omitted for clarity. Right: side view of the host–guest complex.

**Figure 3:**
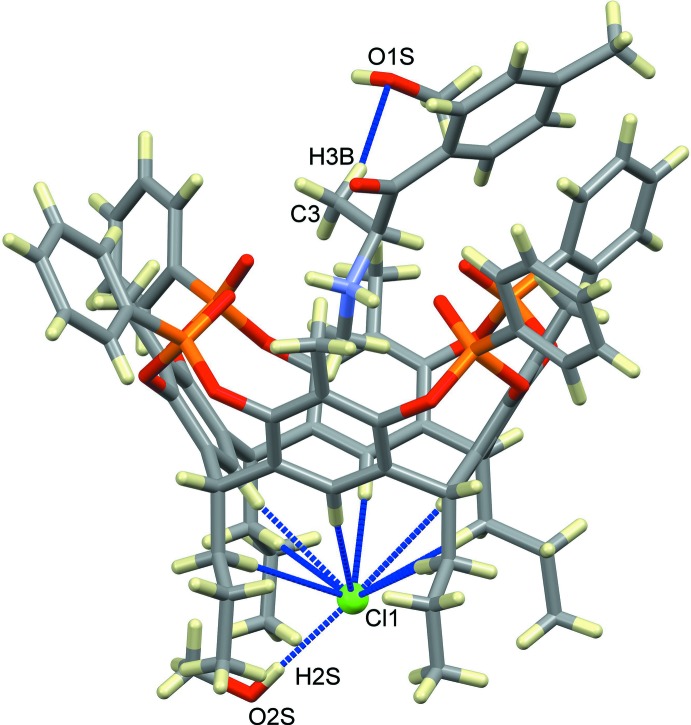
Supra­molecular inter­actions (blue dotted lines) involving the chloride anion (represented as a green sphere) and the disordered methanol lattice mol­ecules.

The host–guest complex (I) of general formula (C_11_H_16_NO)@Tiiii[C_3_H_7_, CH_3_, C_6_H_5_]Cl·CH_3_OH crystallizes in the monoclinic space group *P*21/*c*; its mol­ecular structure is shown in Fig. 1 ▸. It consists of a 1:1 inclusion compound between mephedrone hydro­chloride and a resorcinarene-based tetra­phospho­nate cavitand with the four P=O groups bridging the upper rim all pointing inwards the aromatic cavity. At the lower rim, four propyl chains are present, one of which is disordered over two equivalent positions with occupancy factors of 0.5. For each supra­molecular complex, one lattice methanol mol­ecule is present, disordered over two positions with occupancy factors of 0.665 (6) and 0.335 (6) (see Fig. 3 ▸). The mephedrone cation (C_11_H_16_NO)^+^, which is protonated at the nitro­gen atom N1, is located inside the cavity through the formation of two strong, charge-assisted N—H⋯O hydrogen bonds involving the P=O groups at the upper rim as acceptors (N1—H1*A*⋯O3*A* and N1—H1*B*⋯O3*B*, see Fig. 2 ▸ and Table 1 ▸ for the detailed geom­etrical parameters). The methyl group C1 directly bonded to the amino moiety is located inside the π basic cavity, stabilized *via* a cation⋯π inter­action involving the C1—H1*D* moiety and the aromatic ring C1*B*–C6*B* [C1—H1*D*⋯*Cg*1, 3.672 (7) Å and 145.1°, where C*g*1 is the centroid of the benzene ring]. According to the electrostatic model, the term ‘cation⋯π’ is more appropriate than ‘C—H⋯π’ to describe the inter­actions of N-methyl­ammonium ions (Dougherty, 2013 ▸).] Further stabilization is provided by three C—H_guest_⋯O=P_host_ hydrogen bonds (Fig. 2 ▸ and Table 1 ▸). The distance of C1 from the mean plane passing through the methyl­ene atoms C8*A*, C8*B*, C8*C* and C8*D* of the lower rim is 3.001 (5) Å, which gives a measure of how deeply the guest is inserted inside the cavity (see also the discussion in *Section 5*). The chloride anion is located between the alkyl legs of the cavitand, with a Cl1⋯N1 distance of 7.097 (5) Å, forming numerous C–H⋯Cl inter­actions with the aromatic and methyl­enic hydrogen atoms of the lower rim (see Table 1 ▸), as well as a hydrogen bond with the O2*S*—H2*S* group of the methanol mol­ecule of occupancy factor 0.665 (6) [O2*S*—H2*S*⋯Cl1, 3.105 (5) Å and 168.5 °]. Moreover, the O1*S* atom from the other methanol fraction accepts a hydrogen bond from the methyl group C3 of the mephedrone guest [C3—H3*B*⋯O1*S*, 3.51 (2) Å and 164.3 °].

## Supra­molecular features   

**Figure 4:**
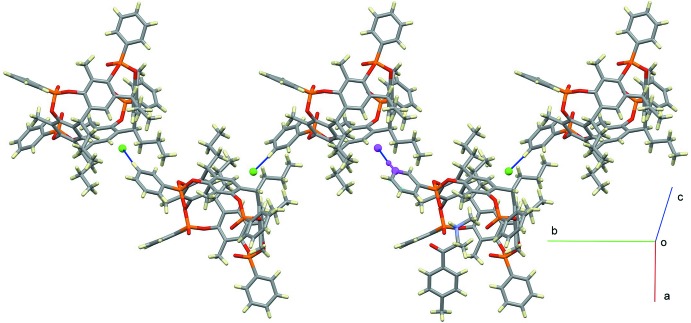
View of the packing of (I) along the *b*-axis direction, mediated by C14*B*—H14*B*⋯Cl^−^ inter­actions (blue lines). The C and H atoms highlighted in purple are in general positions, while the chloride anion is at the symmetry position −*x*, 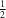 + *y*, 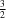 − *z*.

**Figure 5:**
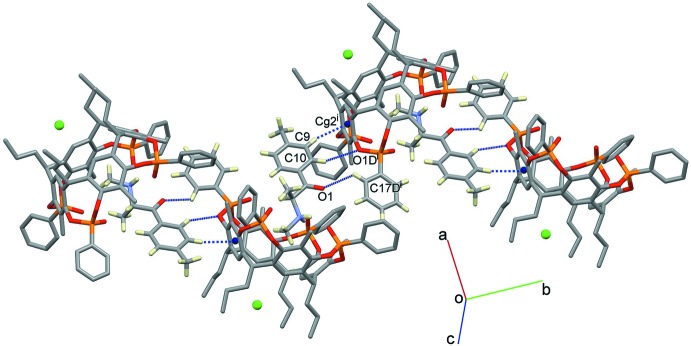
View of the packing of (I) mediated by C—H⋯O and C—H⋯π inter­actions between the guests and adjacent cavitands. Symmetry code: (i) 1 − *x*, 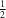 + *y*, 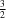 − *z*.

**Figure 6:**
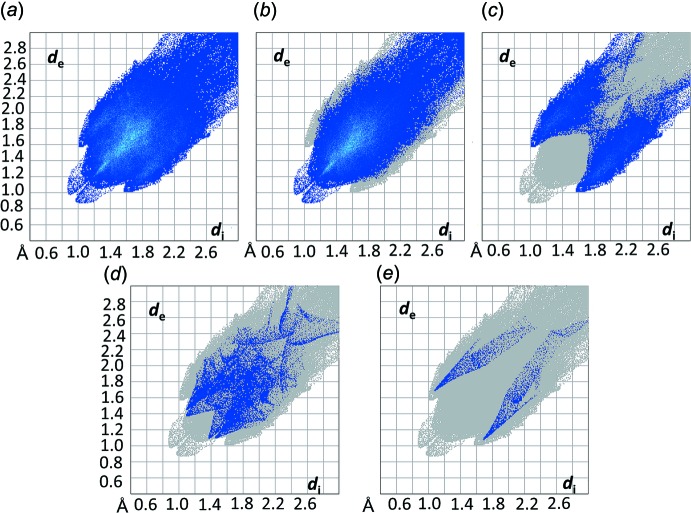
The full two-dimensional fingerprint plot (*a*) and those delineated into H⋯H (*b*), C⋯H/H⋯C (*c*), O⋯H/H⋯O (*d*) and Cl⋯H/H⋯Cl (*e*) contacts for (I).

Besides the supra­molecular inter­actions that yield the 1:1 host–guest complex, mephedrone hydro­chloride also influences the overall packing of the crystal structure, as can be seen from Figs. 4 ▸ and 5 ▸. The chloride anion is responsible for the formation of a supra­molecular chain along the *b*-axis direction through C14*B*—H14*B*⋯Cl^−^(−*x*, 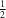 + *y*, 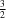 − *z*) contacts involving the phenyl substituents of one of the four phospho­nate groups (Fig. 4 ▸). On the other side, the cationic part of the guest is involved in C—H⋯O and C—H⋯π inter­actions with the phenyl ring bound to the P1*D*=O3*D* group and the aromatic ring C1*D*–C6*D* belonging to the wall of an adjacent cavitand (Fig. 5 ▸ and Table 2 ▸). More precisely, the oxygen atom O1 of the guest acts as a hydrogen-bond acceptor towards the C17*D* ^i^—H17*D* ^i^ group [3.204 (6) Å and 125.0°; symmetry code (i): −*x* + 1, *y* + 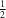, −*z* + 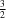], while C9—H9 and C10—H10 act as donors towards the centroid C*g*2^i^ [3.594 (5) Å and 158.7°] and the oxygen atom O1*D* ^i^ [3.555 (4) Å and 151.8°], respectively. These sets of inter­actions can be summarized visually by calculating the two-dimensional fingerprint plots derived from the Hirshfeld surface analysis (Spackman & McKinnon, 2002 ▸; McKinnon *et al.*, 2004 ▸), using the program *Crystal Explorer 17* (Turner *et al.*, 2017 ▸). The overall fingerprint plot for (I) is shown in Fig. 6 ▸ *a* and those delineated in H⋯H (67.8%), C⋯H/H⋯C (23.3%), O⋯H/H⋯O (6.4%) and Cl⋯H/H⋯Cl (1.2%) inter­actions are shown in Fig. 6 ▸ *b*–*e*, respectively (the methanol solvent and the disordered alkyl chain have been omitted from the calculation). Apart from the H⋯H contacts, which probably derive from the inter­actions involving the alkyl chains, the second highest contribution arises from C⋯H/H⋯C contacts (*d*_i_ + *d*_e_ ∼2.58 Å), followed by O⋯H/H⋯O (*d*_i_ + *d*_e_ ∼2.48 Å) and Cl⋯H/H⋯Cl (*d*_i_ + *d*_e_ ∼2.76 Å), all shorter than the respective sums of the van der Waals radii.

## Studies in solution   

**Figure 7:**
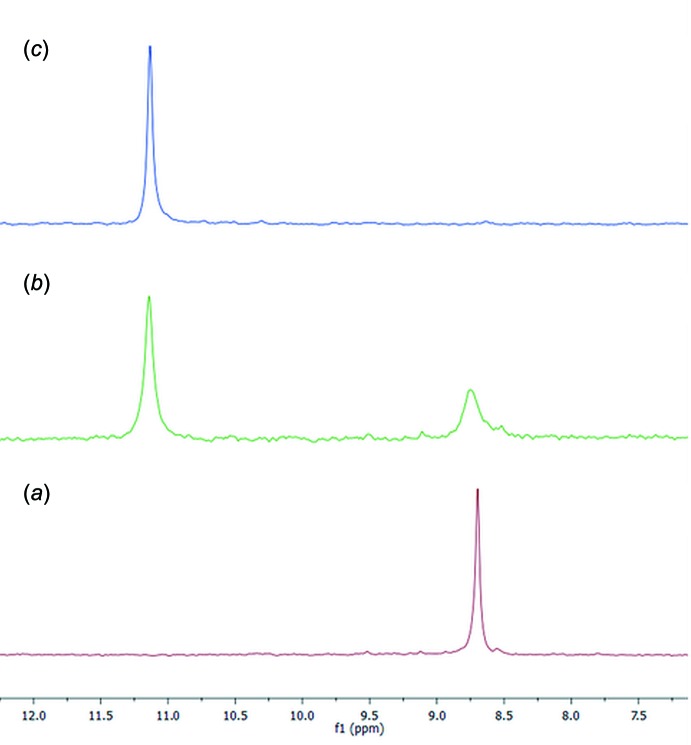
^31^P NMR (162 MHz, MeOD, 253 K) spectra of (*a*) free host Tiiii[C_3_H_7_, CH_3_, Ph]; (*b*) addition of 0.5 equivalent of mephedrone HCl to the host solution; (*c*) addition of 1 equivalent of mephedrone HCl to the host solution.

In solution, complexation was observed both *via* phospho­rous and proton NMR spectroscopy following the shift of the ^31^P signals of the Tiiii[C_3_H_7_, CH_3_, Ph] host and the shift of the ^+^N—CH_3_ protons of the mephedrone hydro­chloride guest. The titration was performed in deuterated methanol at 253 K, in order to be under slow chemical exchange in the NMR time scale and better observe the complexation event. The NMR tube was filled with 0.4 mL of a deuterated methanol solution containing the cavitand (7.5 m*M* concentration). The meph­edrone hydro­chloride titrant solution was prepared by dissolving the guest in 0.1 mL of deuterated methanol (31 m*M*). Two portions (0.5 eq., 48.5 mL) of the titrant were added by syringe to the NMR tube. During the titration, the phospho­rous singlet of the cavitand shifted downfield, from 8.70 (free host) to 11.14 ppm upon addition of one equivalent of the guest (Fig. 7 ▸ *a* and 7*c*), indicating the presence of cation–dipole inter­actions between the ^+^N–CH_3_ and the phospho­nate groups at the upper rim. The addition of 0.5 eq. of guest caused the appearance of two phospho­rous signals at 8.74 and 11.14 ppm related to the free host and to the complex, respectively (Fig. 7 ▸ *b*).

**Figure 8:**
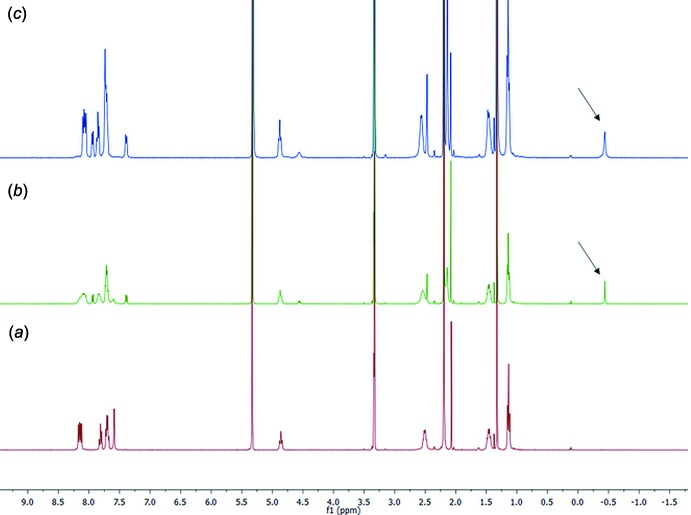
^1^H NMR (400 MHz, MeOD, 253 K) spectra of (*a*) free host Tiiii[C_3_H_7_, CH_3_, Ph]; (*b*) addition of 0.5 equivalent of mephedrone HCl to the host solution; (*c*) addition of 1 equivalent of mephedrone HCl to the host solution. The arrows indicate the up-shift of ^+^N—CH_3_ protons.

In the proton NMR, after the addition of 0.5 equivalent of mephedrone hydro­chloride the diagnostic upfield shift of the guest ^+^N–CH_3_ signals was observed, as expected for the shielding effect caused by its inclusion in the aromatic cavity of the host (Fig. 8 ▸ *b*). After the addition of one equivalent of guest, the ^+^N—CH_3_ singlet appeared still shifted upfield but broadened (Fig. 8 ▸ *c*).

## Database survey   

**Figure 9:**
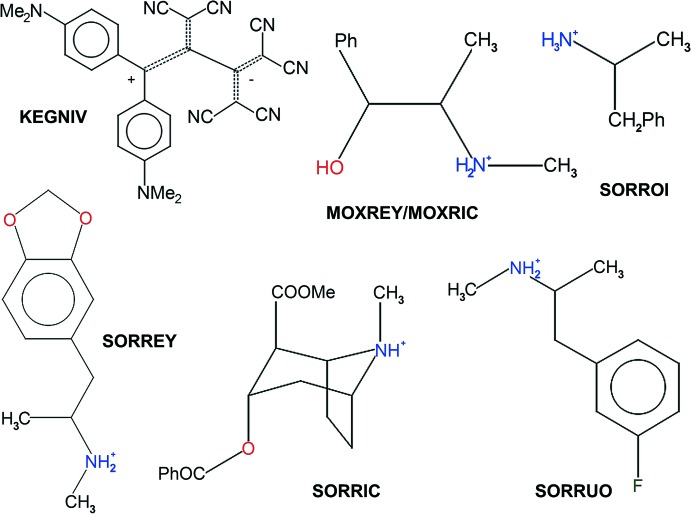
Mol­ecular sketch of the different guests described in the *Database survey.*

As already discussed in *Section 1*, tetra­phospho­nate cavitands of general formula Tiiii[*R*, *R*1, *R*2] (where *R*, *R*1 and *R*2 are the substituents at the lower rim, on the four benzene rings of the cavity, and on the phospho­nate groups, respectively; Pinalli *et al.*, 2004 ▸), are excellent receptors for mol­ecular recognition of neutral and charged guests because of the presence of P=O groups that act as hydrogen-bond acceptors, and of the aromatic cavity that allows the formation of C—H⋯π inter­actions. The substituent *R* at the lower rim can be modified to tune the solubility of the host, to enhance the crystallization process, or to graft the cavity on different surfaces, but does not play any significant role in the recognition process, if not that of inter­acting with the anionic counterpart of a positively charged guest. A search in the Cambridge Structural Database (Version 5.38, update August 2018; Groom *et al.*, 2016 ▸) for a tetra­phospho­nate scaffold without limitations on *R*, *R*1 and *R*2 yielded 82 hits, with the most populated class (44 hits) being the one of general formula Tiiii[H, CH_3_, CH_3_]. The substitution of the alkyl chains with hydrogen atoms favours the formation of crystals, albeit lowering the solubility of the macrocycle, and the methyl group on the phospho­nate moiety generates less steric hindrance than a phenyl one. Besides these general considerations, the most inter­esting structural comparisons with the title compound are to be made with supra­molecular complexes in which the guests are: (i) the zwitterionic species 1,1-di­cyano-2-(di­cyano­meth­yl)-3-(di­cyano­methyl­ene)-4,4-bis[4-(di­methyl­amino)­phen­yl]but-4-ylium-2-ide (KEGNIV; Wu *et al.*, 2012 ▸); (ii) the diasteromeric pair ephedrine and pseudoephedrine hydro­chloride (MOXREY and MOXRIC; Biavardi *et al.*, 2015 ▸); (iii) MDMA, cocaine, amphetamine and 3-fluoro­methamphetamine hydro­chloride (SORREY, SORRIC, SORROI and SORRUO; Biavardi *et al.*, 2014 ▸). A mol­ecular sketch of the guests is reported in Fig. 9 ▸. In the case of KEGNIV, the positive charge of the zwitterionic species is localized on the *N*,*N*-di­methyl­anilino rings, in particular on the NMe_2_ moiety, and that has been demonstrated by the supra­molecular complex formed with Tiiii in which the guest enters the cavity with the positive fragment to form ion–dipole inter­actions with the P=O groups. Ephedrine and pseudoephedrine are complexed by the cavitand *via* a set of supra­molecular contacts very similar to those present in the title compound, that is, hydrogen bonding involving the –NH_2_^+^ fragment as donor and the phospho­nate groups as acceptors, and cation⋯π inter­actions. The distance of the carbon atom of the methyl group inter­acting with the cavity from the mean plane passing through the methyl­ene atoms C8*A*, C8*B*, C8*C* and C8*D* of the lower rim (the labelling is the same as in Fig. 2 ▸) is 3.023 (4) Å for ephedrine, 3.202 (3) Å for the sterically hindered pseudoephedrine and 3.001 (5) Å for (I). This value is of 3.122 (2), 4.104 (4), 2.853 (3) and 2.983 (5) Å for MDMA, cocaine, amphetamine and 3-fluoro­methamphetamine hydro­chloride, respectively, all in good agreement with that of the title compound (cocaine is less included inside the cavity because of its bulky substituents).

## Synthesis and crystallization   

^1^H NMR spectra were obtained using a Bruker AMX-400 (400 MHz) spectrometer. All chemical shifts (δ) were reported in ppm relative to the proton resonances resulting from incomplete deuteration of the NMR solvents. ^31^P NMR spectra were obtained using a Bruker AMX-400 (162 MHz) spectrometer. All chemical shifts (δ) were recorded in ppm relative to external 85% H_3_PO_4_ at 0.00 ppm. The cavitand Tiiii[C_3_H_7_, CH_3_, C_6_H_5_] was prepared following published procedures (Biavardi *et al.*, 2008 ▸). Mephedrone hydro­chloride in its racemic form was purchased from SALAR SpA (Italy) and used as received without further purification.

(C_11_H_16_NO)@Tiiii[C_3_H_7_, CH_3_, C_6_H_5_]Cl·CH_3_OH was obtained by mixing a methanol solution of Tiiii[C_3_H_7_, CH_3_, C_6_H_5_] (1 eq.) with a di­chloro­methane solution of C_11_H_16_NOCl (1 eq.). The mixture was left to evaporate to yield colourless single crystals of the 1:1 complex which were suitable for X-ray diffraction analysis.

## Refinement   

Crystal data, data collection and structure refinement details are summarized in Table 2 ▸. The H atoms bound to C, N and O were placed in calculated positions and refined isotropically using a riding model with C—H ranging from 0.95 to 1.00 Å, N—H = 0.91 Å, O—H = 0.98 Å and *U*iso(H) set to 1.2–1.5*U*eq(C/N/O). For each cavitand:guest complex, a methanol solvent mol­ecule was located in the difference-Fourier map, disordered over two positions with occupancy factors of 0.665 (6) and 0.335 (6). One of the four alkyl chains of the cavitand was also found to be disordered over two equivalent positions with occupancy factors of 0.5, and the relative carbon atoms were refined isotropically. Four reflections showing poor agreement (031, 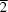
31, 020 and 231) were omitted from the final refinement.

## Supplementary Material

Crystal structure: contains datablock(s) I. DOI: 10.1107/S2056989019001464/ex2018sup1.cif

Structure factors: contains datablock(s) I. DOI: 10.1107/S2056989019001464/ex2018Isup2.hkl

CCDC reference: 1893628

Additional supporting information:  crystallographic information; 3D view; checkCIF report

## Figures and Tables

**Table 1:** Hydrogen-bond geometry (Å, °) C*g*1 and C*g*2 are the centroids of the rings C1*B*–C6*B* and C1*D*–C6*D*, respectively.

*D*—H⋯*A*	*D*—H	H⋯*A*	*D*⋯*A*	*D*—H⋯*A*
N1—H1*A*⋯O3*A*	0.91	1.91	2.773 (5)	157
N1—H1*B*⋯O3*B*	0.91	1.99	2.841 (5)	155
C2—H2⋯O3*D*	1.00	2.25	3.140 (6)	148
C3—H3*A*⋯O3*C*	0.98	2.49	3.351 (7)	147
C3—H3*B*⋯O1*S*	0.98	2.55	3.51 (2)	164
O2*S*—H2*S*⋯Cl1	0.84	2.28	3.105 (5)	169
C1*A*—H1*A*1⋯Cl1	0.95	2.91	3.847 (4)	170
C1*B*—H1*B*1⋯Cl1	0.95	2.93	3.870 (5)	170
C1*C*—H1*C*1⋯Cl1	0.95	2.95	3.888 (5)	170
C1*D*—H1*D*1⋯Cl1	0.95	2.85	3.782 (5)	168
C9*A*—H9*A*1⋯Cl1	0.99	2.76	3.738 (5)	172
C9*B*—H9*B*2⋯Cl1	0.99	2.88	3.870 (4)	175
C9*C*—H9*C*1⋯Cl1	0.99	2.71	3.701 (5)	175
C9*D*—H9*D*1⋯Cl1	0.99	2.85	3.838 (5)	178
C1—H1*D*⋯C*g*1	0.98	2.83	3.672 (7)	145
C17*D* ^i^—H17*D* ^i^⋯O1	0.95	2.56	3.204 (6)	125
C10—H10⋯O1*D* ^i^	0.95	2.69	3.555 (4)	152
C9—H9⋯C*g*2^i^	0.95	2.69	3.594 (5)	159
C14*B*—H14*B*⋯Cl1^ii^	0.95	2.89	3.697 (6)	143

Symmetry codes: (i) 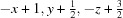; (ii) 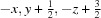.

**Table 2:** Experimental details

Crystal data	
Chemical formula	C_11_H_16_NO^+^·Cl^−^·C_68_H_68_O_12_P_4_·CH_4_O
*M*_r_	1446.84
Crystal system, space group	Monoclinic, *P*2_1_/*c*
Temperature (K)	190
*a*, *b*, *c* (Å)	17.5353 (8), 22.4798 (9), 21.2031 (9)
β (°)	109.455 (1)
*V* (Å^3^)	7880.8 (6)
*Z*	4
Radiation type	Mo *K*α
μ (mm^−1^)	0.19
Crystal size (mm)	0.13 × 0.10 × 0.08

Data collection	
Diffractometer	Bruker APEXII CCD area-detector
Absorption correction	Multi-scan (*SADABS*; Bruker, 2008 ▸)
*T*_min_, *T*_max_	0.634, 0.745
No. of measured, independent and observed [*I* > 2σ(*I*)] reflections	89697, 15077, 9535
*R*_int_	0.073
(sin θ/λ)_max_ (Å^−1^)	0.612

Refinement	
*R*[*F* ^2^ > 2σ(*F* ^2^)], *wR*(*F* ^2^), *S*	0.077, 0.275, 1.02
No. of reflections	15077
No. of parameters	929
No. of restraints	2
H-atom treatment	H-atom parameters constrained
Δρ_max_, Δρ_min_ (e Å^−3^)	1.78, −0.52

Computer programs: *APEX2* and *SAINT* (Bruker, 2008 ▸), *SIR97* (Altomare *et al.*, 1999 ▸), *SHELXL2014/7* (Sheldrick, 2015 ▸), *Mercury* (Macrae *et al.*, 2006 ▸), *WinGX* (Farrugia, 2012 ▸), *PARST* (Nardelli, 1995 ▸) and *publCIF* (Westrip, 2010 ▸).

## supplementary crystallographic information

### Crystal data

**Table:**

C_11_H_16_NO^+^·Cl^−^·C_68_H_68_O_12_P_4_·CH_4_O	*F*(000) = 3056
*M**_r_* = 1446.84	*D*_x_ = 1.219 Mg m^−^^3^
Monoclinic, *P*2_1_/*c*	Mo *K*α radiation, λ = 0.71069 Å
*a* = 17.5353 (8) Å	Cell parameters from 2781 reflections
*b* = 22.4798 (9) Å	θ = 1.4–25.8°
*c* = 21.2031 (9) Å	µ = 0.19 mm^−^^1^
β = 109.455 (1)°	*T* = 190 K
*V* = 7880.8 (6) Å^3^	Prismatic, colourless
*Z* = 4	0.13 × 0.10 × 0.08 mm

### Data collection

**Table:**

Bruker APEXII CCD area-detector diffractometer	15077 independent reflections
Radiation source: fine-focus sealed tube	9535 reflections with *I* > 2σ(*I*)
Graphite monochromator	*R*_int_ = 0.073
ω–scan	θ_max_ = 25.8°, θ_min_ = 1.4°
Absorption correction: multi-scan (SADABS; Bruker, 2008)	*h* = −21→21
*T*_min_ = 0.634, *T*_max_ = 0.745	*k* = −27→27
89697 measured reflections	*l* = −25→25

### Refinement

**Table:**

Refinement on *F*^2^	2 restraints
Least-squares matrix: full	Hydrogen site location: inferred from neighbouring sites
*R*[*F*^2^ > 2σ(*F*^2^)] = 0.077	H-atom parameters constrained
*wR*(*F*^2^) = 0.275	*w* = 1/[σ^2^(*F*_o_^2^) + (0.1708*P*)^2^ + 6.7275*P*] where *P* = (*F*_o_^2^ + 2*F*_c_^2^)/3
*S* = 1.02	(Δ/σ)_max_ = 0.001
15077 reflections	Δρ_max_ = 1.78 e Å^−^^3^
929 parameters	Δρ_min_ = −0.52 e Å^−^^3^

### Special details

**Table:**

Geometry. All esds (except the esd in the dihedral angle between two l.s. planes) are estimated using the full covariance matrix. The cell esds are taken into account individually in the estimation of esds in distances, angles and torsion angles; correlations between esds in cell parameters are only used when they are defined by crystal symmetry. An approximate (isotropic) treatment of cell esds is used for estimating esds involving l.s. planes.

### Fractional atomic coordinates and isotropic or equivalent isotropic displacement parameters (Å^2^)

**Table:**

	*x*	*y*	*z*	*U*_iso_*/*U*_eq_	Occ. (<1)
Cl1	0.08550 (8)	−0.09484 (7)	0.83375 (7)	0.0629 (4)	
N1	0.3301 (2)	0.10053 (18)	0.7202 (2)	0.0554 (10)	
H1A	0.3375	0.1203	0.7593	0.066*	
H1B	0.2979	0.1234	0.6864	0.066*	
O1	0.4139 (3)	0.19702 (17)	0.7037 (2)	0.0786 (12)	
C1	0.2883 (3)	0.0432 (2)	0.7216 (3)	0.0559 (12)	
H1C	0.2744	0.0239	0.6778	0.084*	
H1D	0.2389	0.0508	0.7322	0.084*	
H1E	0.3242	0.0172	0.7559	0.084*	
C2	0.4103 (3)	0.0926 (2)	0.7107 (3)	0.0559 (12)	
H2	0.4429	0.0636	0.7448	0.067*	
C3	0.4012 (5)	0.0691 (3)	0.6436 (4)	0.094 (2)	
H3A	0.3662	0.0340	0.6347	0.141*	
H3B	0.4545	0.0581	0.6417	0.141*	
H3C	0.3770	0.0997	0.6099	0.141*	
C4	0.4542 (3)	0.1522 (2)	0.7228 (3)	0.0578 (13)	
C5	0.5426 (3)	0.1539 (2)	0.7557 (3)	0.0556 (12)	
C6	0.5884 (4)	0.1030 (2)	0.7777 (3)	0.0725 (16)	
H6	0.5632	0.0651	0.7702	0.087*	
C7	0.6700 (4)	0.1074 (3)	0.8101 (4)	0.086 (2)	
H7	0.7007	0.0721	0.8242	0.103*	
C8	0.7091 (3)	0.1621 (3)	0.8230 (3)	0.0669 (15)	
C9	0.6637 (4)	0.2117 (3)	0.7986 (3)	0.0778 (17)	
H9	0.6892	0.2495	0.8048	0.093*	
C10	0.5824 (4)	0.2080 (2)	0.7656 (3)	0.0743 (17)	
H10	0.5526	0.2433	0.7491	0.089*	
C11	0.7975 (4)	0.1679 (3)	0.8644 (4)	0.097 (2)	
H11A	0.8306	0.1625	0.8356	0.145*	
H11B	0.8117	0.1375	0.8995	0.145*	
H11C	0.8074	0.2075	0.8849	0.145*	
P1A	0.38511 (7)	0.16364 (5)	0.90714 (5)	0.0380 (3)	
O1A	0.40936 (17)	0.10664 (12)	0.95332 (13)	0.0384 (6)	
O2A	0.29380 (17)	0.17777 (13)	0.90051 (14)	0.0433 (7)	
O3A	0.39674 (18)	0.15745 (13)	0.84232 (14)	0.0449 (7)	
P1B	0.10885 (7)	0.17333 (5)	0.61816 (6)	0.0407 (3)	
O1B	0.08989 (17)	0.18649 (13)	0.68474 (15)	0.0427 (7)	
O2B	0.04768 (17)	0.12187 (12)	0.58070 (14)	0.0407 (7)	
O3B	0.19354 (18)	0.15819 (13)	0.62903 (16)	0.0475 (7)	
P1C	0.21929 (7)	−0.08274 (5)	0.52556 (6)	0.0417 (3)	
O1C	0.12856 (17)	−0.06563 (13)	0.51623 (14)	0.0404 (7)	
O2C	0.23930 (17)	−0.13912 (13)	0.57372 (14)	0.0417 (7)	
O3C	0.2765 (2)	−0.03344 (15)	0.54850 (17)	0.0556 (8)	
P1D	0.50908 (6)	−0.08324 (5)	0.81214 (5)	0.0354 (3)	
O1D	0.45599 (16)	−0.14052 (12)	0.78262 (14)	0.0375 (6)	
O2D	0.50278 (16)	−0.07416 (12)	0.88471 (13)	0.0355 (6)	
O3D	0.48583 (18)	−0.03031 (13)	0.76989 (15)	0.0461 (7)	
C10D	0.3303 (3)	−0.1689 (2)	0.9668 (2)	0.0537 (12)	
H10K	0.3883	−0.1796	0.9806	0.064*	
H10J	0.3263	−0.1285	0.9842	0.064*	
C2A	0.3159 (2)	0.02458 (18)	0.91576 (19)	0.0362 (9)	
C3A	0.3928 (2)	0.04831 (18)	0.92624 (19)	0.0355 (9)	
C4A	0.4552 (2)	0.01803 (17)	0.91511 (18)	0.0336 (9)	
C5A	0.4385 (2)	−0.04078 (17)	0.89356 (19)	0.0342 (9)	
C6A	0.3637 (2)	−0.06805 (17)	0.88228 (18)	0.0331 (9)	
C7A	0.5366 (2)	0.04572 (19)	0.9266 (2)	0.0414 (10)	
H7A1	0.5315	0.0781	0.8946	0.062*	
H7A2	0.5740	0.0156	0.9206	0.062*	
H7A3	0.5575	0.0615	0.9723	0.062*	
C8A	0.2484 (2)	0.0611 (2)	0.9277 (2)	0.0403 (10)	
H8A	0.2747	0.0916	0.9625	0.048*	
C9A	0.1926 (3)	0.0241 (3)	0.9539 (2)	0.0570 (13)	0.5
H9A1	0.1650	−0.0048	0.9184	0.068*	0.5
H9A2	0.2276	0.0007	0.9922	0.068*	0.5
C10A	0.1322 (6)	0.0507 (4)	0.9750 (5)	0.049 (2)*	0.5
H10A	0.0934	0.0712	0.9361	0.059*	0.5
H10B	0.1581	0.0815	1.0087	0.059*	0.5
C11A	0.0858 (8)	0.0104 (6)	1.0041 (7)	0.079 (3)*	0.5
H11D	0.0636	−0.0226	0.9731	0.119*	0.5
H11E	0.0415	0.0324	1.0117	0.119*	0.5
H11F	0.1217	−0.0055	1.0467	0.119*	0.5
C9E	0.1926 (3)	0.0241 (3)	0.9539 (2)	0.0570 (13)	0.5
H9E1	0.1444	0.0483	0.9512	0.068*	0.5
H9E2	0.1738	−0.0108	0.9242	0.068*	0.5
C10E	0.2270 (7)	0.0042 (5)	1.0187 (5)	0.060 (3)*	0.5
H10C	0.2530	0.0378	1.0481	0.072*	0.5
H10D	0.2692	−0.0258	1.0207	0.072*	0.5
C11E	0.1624 (9)	−0.0237 (7)	1.0435 (7)	0.093 (4)*	0.5
H11G	0.1221	0.0065	1.0435	0.139*	0.5
H11H	0.1879	−0.0389	1.0890	0.139*	0.5
H11I	0.1359	−0.0564	1.0138	0.139*	0.5
C12A	0.4380 (3)	0.22140 (18)	0.9610 (2)	0.0413 (10)	
C13A	0.4582 (4)	0.2721 (3)	0.9337 (3)	0.0745 (18)	
H13A	0.4484	0.2742	0.8870	0.089*	
C14A	0.4927 (5)	0.3200 (3)	0.9744 (4)	0.087 (2)	
H14A	0.5028	0.3561	0.9552	0.105*	
C15A	0.5122 (4)	0.3151 (3)	1.0423 (3)	0.0742 (17)	
H15A	0.5395	0.3468	1.0705	0.089*	
C16A	0.4927 (5)	0.2649 (3)	1.0695 (3)	0.084 (2)	
H16A	0.5048	0.2623	1.1166	0.101*	
C17A	0.4551 (4)	0.2178 (2)	1.0288 (3)	0.0710 (17)	
H17A	0.4412	0.1830	1.0480	0.085*	
C1B	0.1377 (2)	0.06971 (19)	0.8132 (2)	0.0378 (9)	
H1B1	0.1192	0.0314	0.8203	0.045*	
C2B	0.0980 (2)	0.09968 (19)	0.7532 (2)	0.0383 (9)	
C3B	0.1283 (2)	0.15481 (19)	0.7442 (2)	0.0401 (10)	
C4B	0.1936 (3)	0.18243 (19)	0.7916 (2)	0.0433 (10)	
C5B	0.2294 (2)	0.1501 (2)	0.8505 (2)	0.0409 (10)	
C6B	0.2035 (2)	0.09445 (19)	0.8628 (2)	0.0384 (9)	
C7B	0.2230 (3)	0.2430 (2)	0.7796 (3)	0.0528 (12)	
H7B1	0.2473	0.2634	0.8226	0.079*	
H7B2	0.1773	0.2665	0.7513	0.079*	
H7B3	0.2635	0.2387	0.7574	0.079*	
C8B	0.0260 (2)	0.07312 (19)	0.7000 (2)	0.0389 (9)	
H8B	−0.0063	0.1069	0.6737	0.047*	
C9B	−0.0300 (3)	0.0374 (2)	0.7276 (2)	0.0449 (10)	
H9B1	−0.0763	0.0228	0.6896	0.054*	
H9B2	−0.0003	0.0023	0.7518	0.054*	
C10B	−0.0624 (3)	0.0726 (2)	0.7747 (3)	0.0586 (13)	
H10E	−0.0903	0.1089	0.7518	0.070*	
H10F	−0.0170	0.0851	0.8146	0.070*	
C11B	−0.1216 (5)	0.0344 (3)	0.7964 (4)	0.100 (2)	
H11L	−0.1605	0.0158	0.7569	0.150*	
H11M	−0.1506	0.0596	0.8187	0.150*	
H11N	−0.0917	0.0035	0.8274	0.150*	
C12B	0.0698 (3)	0.23712 (19)	0.5690 (2)	0.0447 (10)	
C13B	0.0186 (3)	0.2771 (2)	0.5852 (3)	0.0543 (12)	
H13B	0.0028	0.2702	0.6232	0.065*	
C14B	−0.0093 (4)	0.3272 (2)	0.5457 (3)	0.0684 (15)	
H14B	−0.0448	0.3543	0.5563	0.082*	
C15B	0.0146 (4)	0.3371 (3)	0.4913 (3)	0.0713 (16)	
H15B	−0.0050	0.3712	0.4642	0.086*	
C16B	0.0660 (4)	0.2989 (3)	0.4751 (3)	0.0773 (18)	
H16B	0.0826	0.3068	0.4376	0.093*	
C17B	0.0939 (4)	0.2482 (2)	0.5142 (3)	0.0661 (15)	
H17B	0.1294	0.2214	0.5032	0.079*	
C1C	0.0744 (2)	−0.02396 (18)	0.6637 (2)	0.0362 (9)	
H1C1	0.0697	−0.0417	0.7028	0.043*	
C2C	0.0996 (2)	−0.05894 (18)	0.6198 (2)	0.0362 (9)	
C3C	0.1080 (2)	−0.03113 (18)	0.56427 (19)	0.0354 (9)	
C4C	0.0937 (2)	0.02916 (19)	0.5505 (2)	0.0384 (9)	
C5C	0.0667 (2)	0.06113 (18)	0.5952 (2)	0.0362 (9)	
C6C	0.0560 (2)	0.03618 (18)	0.6517 (2)	0.0367 (9)	
C7C	0.1049 (3)	0.0577 (2)	0.4899 (2)	0.0440 (10)	
H7C1	0.0730	0.0943	0.4790	0.066*	
H7C2	0.0868	0.0302	0.4520	0.066*	
H7C3	0.1622	0.0671	0.4994	0.066*	
C8C	0.1184 (2)	−0.12493 (18)	0.6345 (2)	0.0367 (9)	
H8C	0.1102	−0.1449	0.5907	0.044*	
C9C	0.0606 (3)	−0.15446 (19)	0.6653 (2)	0.0437 (10)	
H9C1	0.0689	−0.1364	0.7097	0.052*	
H9C2	0.0043	−0.1457	0.6368	0.052*	
C10C	0.0702 (3)	−0.2207 (2)	0.6736 (3)	0.0529 (12)	
H10H	0.1250	−0.2298	0.7048	0.063*	
H10G	0.0649	−0.2389	0.6299	0.063*	
C11C	0.0071 (3)	−0.2479 (2)	0.7006 (3)	0.0671 (15)	
H11O	0.0153	−0.2327	0.7457	0.101*	
H11P	0.0128	−0.2913	0.7023	0.101*	
H11Q	−0.0473	−0.2372	0.6711	0.101*	
C12C	0.2126 (3)	−0.1168 (2)	0.4482 (2)	0.0498 (11)	
C13C	0.2679 (4)	−0.1017 (3)	0.4170 (3)	0.0715 (16)	
H13C	0.3060	−0.0708	0.4343	0.086*	
C14C	0.2664 (4)	−0.1332 (3)	0.3590 (3)	0.0816 (19)	
H14C	0.3040	−0.1235	0.3370	0.098*	
C15C	0.2118 (4)	−0.1771 (3)	0.3345 (3)	0.0738 (18)	
H15C	0.2117	−0.1980	0.2955	0.089*	
C16C	0.1580 (4)	−0.1916 (3)	0.3643 (3)	0.0727 (16)	
H16C	0.1198	−0.2223	0.3460	0.087*	
C17C	0.1574 (3)	−0.1621 (2)	0.4216 (2)	0.0570 (13)	
H17C	0.1193	−0.1729	0.4427	0.068*	
C1D	0.2356 (2)	−0.13048 (17)	0.7459 (2)	0.0361 (9)	
H1D1	0.1975	−0.1283	0.7689	0.043*	
C2D	0.3179 (2)	−0.13337 (17)	0.7825 (2)	0.0346 (9)	
C3D	0.3721 (2)	−0.13563 (17)	0.7472 (2)	0.0370 (9)	
C4D	0.3488 (2)	−0.13616 (19)	0.6780 (2)	0.0384 (9)	
C5D	0.2659 (3)	−0.13406 (18)	0.6444 (2)	0.0385 (9)	
C6D	0.2079 (2)	−0.13078 (17)	0.6756 (2)	0.0366 (9)	
C7D	0.4084 (3)	−0.1409 (2)	0.6415 (2)	0.0512 (12)	
H7D1	0.3815	−0.1572	0.5967	0.077*	
H7D2	0.4529	−0.1672	0.6662	0.077*	
H7D3	0.4300	−0.1013	0.6376	0.077*	
C8D	0.3491 (3)	−0.13214 (18)	0.8587 (2)	0.0370 (9)	
H8D	0.4032	−0.1521	0.8732	0.044*	
C9D	0.2954 (3)	−0.1677 (2)	0.8902 (2)	0.0447 (10)	
H9D1	0.2408	−0.1497	0.8762	0.054*	
H9D2	0.2899	−0.2090	0.8731	0.054*	
C1A	0.3029 (2)	−0.03379 (19)	0.89266 (19)	0.0373 (9)	
H1A1	0.2507	−0.0508	0.8837	0.045*	
C11D	0.2872 (5)	−0.2126 (3)	0.9978 (3)	0.088 (2)	
H11R	0.2305	−0.2006	0.9869	0.133*	
H11S	0.3135	−0.2128	1.0465	0.133*	
H11T	0.2899	−0.2525	0.9800	0.133*	
C12D	0.6095 (3)	−0.11008 (19)	0.8291 (2)	0.0403 (9)	
C13D	0.6686 (4)	−0.0701 (3)	0.8263 (4)	0.084 (2)	
H13D	0.6555	−0.0293	0.8174	0.101*	
C14D	0.7447 (4)	−0.0892 (3)	0.8361 (5)	0.100 (3)	
H14D	0.7837	−0.0619	0.8312	0.120*	
C15D	0.7671 (3)	−0.1469 (3)	0.8528 (3)	0.0649 (15)	
H15D	0.8217	−0.1590	0.8629	0.078*	
C16D	0.7105 (3)	−0.1858 (3)	0.8548 (3)	0.0718 (16)	
H16D	0.7248	−0.2262	0.8655	0.086*	
C17D	0.6309 (3)	−0.1678 (2)	0.8413 (3)	0.0604 (14)	
H17D	0.5909	−0.1965	0.8406	0.072*	
O1S	0.5707 (10)	0.0241 (7)	0.6032 (8)	0.103 (5)*	0.335 (6)
H1S	0.5450	0.0115	0.5647	0.154*	0.335 (6)
C1S	0.6098 (12)	−0.0253 (8)	0.6455 (11)	0.105 (8)	0.335 (6)
H1S1	0.6675	−0.0167	0.6666	0.158*	0.335 (6)
H1S2	0.6033	−0.0615	0.6184	0.158*	0.335 (6)
H1S3	0.5851	−0.0313	0.6802	0.158*	0.335 (6)
O2S	−0.0947 (3)	−0.1331 (3)	0.7768 (3)	0.0699 (19)	0.665 (6)
H2S	−0.0485	−0.1178	0.7930	0.105*	0.665 (6)
C2S	−0.1305 (5)	−0.1142 (4)	0.7091 (4)	0.075 (3)	0.665 (6)
H2S1	−0.1836	−0.0967	0.7029	0.113*	0.665 (6)
H2S2	−0.1367	−0.1485	0.6793	0.113*	0.665 (6)
H2S3	−0.0956	−0.0845	0.6985	0.113*	0.665 (6)

### Atomic displacement parameters (Å^2^)

**Table:**

	*U*^11^	*U*^22^	*U*^33^	*U*^12^	*U*^13^	*U*^23^
Cl1	0.0503 (7)	0.0783 (9)	0.0617 (8)	−0.0120 (6)	0.0208 (6)	−0.0002 (7)
N1	0.052 (2)	0.053 (2)	0.058 (2)	0.0033 (19)	0.013 (2)	−0.0020 (19)
O1	0.083 (3)	0.053 (2)	0.095 (3)	0.008 (2)	0.024 (2)	0.013 (2)
C1	0.046 (3)	0.051 (3)	0.072 (3)	0.000 (2)	0.021 (2)	0.002 (2)
C2	0.053 (3)	0.050 (3)	0.066 (3)	0.004 (2)	0.021 (2)	−0.008 (2)
C3	0.107 (6)	0.081 (5)	0.101 (5)	−0.029 (4)	0.042 (4)	−0.035 (4)
C4	0.071 (3)	0.045 (3)	0.061 (3)	0.007 (3)	0.026 (3)	0.007 (2)
C5	0.061 (3)	0.047 (3)	0.062 (3)	−0.002 (2)	0.025 (3)	0.000 (2)
C6	0.067 (4)	0.042 (3)	0.111 (5)	−0.002 (3)	0.035 (3)	0.000 (3)
C7	0.052 (3)	0.054 (3)	0.147 (6)	0.001 (3)	0.027 (4)	0.014 (4)
C8	0.062 (3)	0.059 (3)	0.085 (4)	−0.008 (3)	0.031 (3)	−0.002 (3)
C9	0.079 (4)	0.054 (3)	0.092 (4)	−0.016 (3)	0.017 (3)	−0.001 (3)
C10	0.081 (4)	0.041 (3)	0.088 (4)	−0.009 (3)	0.010 (3)	−0.001 (3)
C11	0.063 (4)	0.083 (5)	0.145 (7)	−0.019 (3)	0.035 (4)	−0.011 (4)
P1A	0.0383 (6)	0.0354 (6)	0.0377 (6)	−0.0013 (5)	0.0090 (5)	−0.0070 (4)
O1A	0.0392 (15)	0.0342 (15)	0.0383 (15)	−0.0018 (12)	0.0082 (12)	−0.0061 (12)
O2A	0.0382 (16)	0.0421 (17)	0.0453 (17)	0.0010 (13)	0.0081 (13)	−0.0092 (13)
O3A	0.0487 (18)	0.0447 (17)	0.0395 (16)	−0.0002 (14)	0.0122 (13)	−0.0051 (13)
P1B	0.0378 (6)	0.0340 (6)	0.0476 (6)	0.0005 (5)	0.0107 (5)	0.0004 (5)
O1B	0.0407 (16)	0.0365 (15)	0.0467 (17)	0.0025 (13)	0.0089 (13)	−0.0001 (13)
O2B	0.0400 (16)	0.0313 (15)	0.0455 (16)	0.0029 (12)	0.0073 (13)	0.0007 (12)
O3B	0.0395 (16)	0.0416 (17)	0.0597 (19)	0.0006 (13)	0.0143 (14)	0.0013 (14)
P1C	0.0397 (6)	0.0435 (6)	0.0386 (6)	0.0003 (5)	0.0085 (5)	−0.0026 (5)
O1C	0.0395 (16)	0.0388 (16)	0.0377 (15)	0.0004 (13)	0.0061 (12)	−0.0054 (12)
O2C	0.0415 (16)	0.0445 (17)	0.0347 (15)	0.0036 (13)	0.0070 (12)	−0.0047 (13)
O3C	0.0477 (18)	0.054 (2)	0.061 (2)	−0.0095 (16)	0.0119 (16)	−0.0028 (16)
P1D	0.0342 (5)	0.0300 (5)	0.0391 (6)	0.0011 (4)	0.0086 (4)	0.0033 (4)
O1D	0.0314 (14)	0.0350 (15)	0.0401 (15)	0.0029 (12)	0.0040 (12)	0.0005 (12)
O2D	0.0322 (14)	0.0331 (14)	0.0373 (15)	0.0029 (11)	0.0065 (12)	0.0032 (11)
O3D	0.0518 (18)	0.0388 (16)	0.0449 (17)	0.0049 (14)	0.0123 (14)	0.0078 (13)
C10D	0.064 (3)	0.050 (3)	0.046 (3)	−0.012 (2)	0.018 (2)	0.007 (2)
C2A	0.037 (2)	0.040 (2)	0.0301 (19)	−0.0011 (18)	0.0091 (17)	0.0005 (17)
C3A	0.037 (2)	0.033 (2)	0.034 (2)	−0.0050 (17)	0.0080 (17)	−0.0011 (16)
C4A	0.036 (2)	0.033 (2)	0.0300 (19)	−0.0030 (17)	0.0079 (16)	0.0005 (16)
C5A	0.037 (2)	0.033 (2)	0.0309 (19)	0.0009 (17)	0.0096 (17)	0.0044 (16)
C6A	0.035 (2)	0.033 (2)	0.0283 (19)	−0.0050 (17)	0.0060 (16)	0.0031 (16)
C7A	0.036 (2)	0.038 (2)	0.049 (2)	−0.0051 (18)	0.0124 (19)	−0.0031 (19)
C8A	0.034 (2)	0.051 (3)	0.037 (2)	−0.0044 (19)	0.0141 (18)	−0.0066 (19)
C9A	0.055 (3)	0.078 (4)	0.043 (3)	−0.011 (3)	0.023 (2)	−0.007 (2)
C9E	0.055 (3)	0.078 (4)	0.043 (3)	−0.011 (3)	0.023 (2)	−0.007 (2)
C12A	0.040 (2)	0.033 (2)	0.049 (2)	−0.0010 (18)	0.0126 (19)	−0.0100 (19)
C13A	0.112 (5)	0.067 (4)	0.061 (3)	−0.040 (3)	0.051 (3)	−0.025 (3)
C14A	0.124 (6)	0.064 (4)	0.099 (5)	−0.049 (4)	0.071 (5)	−0.030 (3)
C15A	0.078 (4)	0.057 (3)	0.088 (4)	−0.018 (3)	0.028 (3)	−0.035 (3)
C16A	0.124 (6)	0.060 (4)	0.052 (3)	−0.013 (4)	0.007 (3)	−0.018 (3)
C17A	0.109 (5)	0.043 (3)	0.044 (3)	−0.005 (3)	0.004 (3)	−0.002 (2)
C1B	0.033 (2)	0.041 (2)	0.043 (2)	0.0011 (17)	0.0161 (18)	−0.0032 (18)
C2B	0.032 (2)	0.043 (2)	0.042 (2)	0.0030 (18)	0.0134 (18)	−0.0039 (18)
C3B	0.035 (2)	0.041 (2)	0.042 (2)	0.0079 (18)	0.0099 (18)	−0.0019 (19)
C4B	0.038 (2)	0.037 (2)	0.053 (3)	0.0032 (18)	0.012 (2)	−0.006 (2)
C5B	0.033 (2)	0.047 (2)	0.040 (2)	0.0035 (19)	0.0090 (18)	−0.0120 (19)
C6B	0.034 (2)	0.043 (2)	0.039 (2)	0.0030 (18)	0.0132 (18)	−0.0050 (18)
C7B	0.053 (3)	0.034 (2)	0.064 (3)	0.000 (2)	0.009 (2)	−0.003 (2)
C8B	0.033 (2)	0.036 (2)	0.047 (2)	0.0015 (17)	0.0130 (18)	0.0020 (18)
C9B	0.035 (2)	0.046 (2)	0.053 (3)	0.0001 (19)	0.013 (2)	0.004 (2)
C10B	0.051 (3)	0.057 (3)	0.076 (3)	0.011 (2)	0.031 (3)	0.010 (3)
C11B	0.108 (5)	0.081 (5)	0.146 (7)	−0.001 (4)	0.089 (5)	0.009 (5)
C12B	0.044 (2)	0.036 (2)	0.050 (3)	−0.0016 (19)	0.009 (2)	−0.0021 (19)
C13B	0.053 (3)	0.044 (3)	0.065 (3)	0.007 (2)	0.019 (2)	0.002 (2)
C14B	0.070 (4)	0.053 (3)	0.079 (4)	0.019 (3)	0.021 (3)	0.009 (3)
C15B	0.093 (4)	0.050 (3)	0.059 (3)	0.019 (3)	0.009 (3)	0.010 (3)
C16B	0.119 (5)	0.060 (3)	0.051 (3)	0.014 (4)	0.026 (3)	0.013 (3)
C17B	0.097 (4)	0.044 (3)	0.063 (3)	0.013 (3)	0.034 (3)	0.004 (2)
C1C	0.0279 (19)	0.038 (2)	0.037 (2)	−0.0038 (17)	0.0036 (17)	0.0007 (17)
C2C	0.029 (2)	0.035 (2)	0.040 (2)	−0.0039 (17)	0.0040 (17)	−0.0018 (17)
C3C	0.033 (2)	0.037 (2)	0.032 (2)	−0.0024 (17)	0.0049 (16)	−0.0031 (17)
C4C	0.032 (2)	0.041 (2)	0.036 (2)	−0.0002 (17)	0.0016 (17)	0.0002 (18)
C5C	0.030 (2)	0.033 (2)	0.040 (2)	−0.0019 (16)	0.0035 (17)	0.0012 (17)
C6C	0.028 (2)	0.037 (2)	0.043 (2)	−0.0052 (17)	0.0081 (17)	−0.0042 (18)
C7C	0.046 (2)	0.042 (2)	0.041 (2)	−0.001 (2)	0.011 (2)	0.0046 (19)
C8C	0.035 (2)	0.033 (2)	0.038 (2)	−0.0013 (17)	0.0059 (17)	−0.0033 (17)
C9C	0.037 (2)	0.040 (2)	0.049 (3)	−0.0077 (19)	0.0072 (19)	0.0011 (19)
C10C	0.048 (3)	0.039 (2)	0.067 (3)	−0.003 (2)	0.014 (2)	0.004 (2)
C11C	0.070 (4)	0.049 (3)	0.084 (4)	−0.006 (3)	0.027 (3)	0.012 (3)
C12C	0.050 (3)	0.056 (3)	0.037 (2)	0.012 (2)	0.006 (2)	0.005 (2)
C13C	0.075 (4)	0.090 (4)	0.056 (3)	0.000 (3)	0.030 (3)	0.004 (3)
C14C	0.086 (5)	0.112 (5)	0.059 (4)	0.025 (4)	0.041 (3)	0.009 (4)
C15C	0.079 (4)	0.088 (5)	0.043 (3)	0.031 (4)	0.005 (3)	−0.005 (3)
C16C	0.084 (4)	0.075 (4)	0.050 (3)	0.009 (3)	0.010 (3)	−0.013 (3)
C17C	0.059 (3)	0.060 (3)	0.044 (3)	0.006 (2)	0.006 (2)	−0.008 (2)
C1D	0.037 (2)	0.032 (2)	0.037 (2)	−0.0037 (17)	0.0101 (17)	0.0004 (17)
C2D	0.034 (2)	0.030 (2)	0.037 (2)	−0.0036 (16)	0.0073 (17)	0.0016 (16)
C3D	0.032 (2)	0.030 (2)	0.041 (2)	−0.0013 (17)	0.0025 (17)	0.0007 (17)
C4D	0.037 (2)	0.038 (2)	0.038 (2)	0.0034 (18)	0.0102 (18)	−0.0012 (18)
C5D	0.043 (2)	0.035 (2)	0.034 (2)	0.0042 (18)	0.0080 (18)	0.0009 (17)
C6D	0.037 (2)	0.0263 (19)	0.040 (2)	−0.0029 (17)	0.0041 (17)	−0.0001 (16)
C7D	0.043 (3)	0.062 (3)	0.047 (3)	0.008 (2)	0.013 (2)	−0.005 (2)
C8D	0.040 (2)	0.031 (2)	0.037 (2)	−0.0026 (17)	0.0076 (18)	0.0062 (17)
C9D	0.050 (3)	0.039 (2)	0.042 (2)	−0.013 (2)	0.011 (2)	0.0041 (19)
C1A	0.036 (2)	0.040 (2)	0.034 (2)	−0.0079 (18)	0.0086 (17)	0.0046 (17)
C11D	0.122 (6)	0.089 (5)	0.057 (3)	−0.039 (4)	0.034 (4)	0.009 (3)
C12D	0.037 (2)	0.038 (2)	0.044 (2)	0.0008 (18)	0.0114 (18)	−0.0003 (18)
C13D	0.058 (3)	0.048 (3)	0.160 (7)	0.006 (3)	0.054 (4)	0.025 (4)
C14D	0.058 (4)	0.076 (4)	0.183 (8)	−0.001 (3)	0.061 (5)	0.032 (5)
C15D	0.041 (3)	0.067 (4)	0.087 (4)	0.006 (3)	0.021 (3)	−0.007 (3)
C16D	0.046 (3)	0.051 (3)	0.113 (5)	0.009 (3)	0.019 (3)	0.008 (3)
C17D	0.045 (3)	0.039 (3)	0.095 (4)	−0.001 (2)	0.021 (3)	0.008 (3)
C1S	0.095 (16)	0.099 (17)	0.15 (2)	−0.026 (13)	0.078 (16)	−0.010 (16)
O2S	0.037 (3)	0.080 (4)	0.094 (5)	−0.021 (3)	0.024 (3)	0.001 (3)
C2S	0.057 (5)	0.054 (5)	0.120 (8)	−0.015 (4)	0.035 (5)	−0.010 (5)

### Geometric parameters (Å, º)

**Table:**

N1—C1	1.488 (6)	C3B—C4B	1.393 (6)
N1—C2	1.495 (6)	C4B—C5B	1.400 (6)
N1—H1A	0.9100	C4B—C7B	1.507 (6)
N1—H1B	0.9100	C5B—C6B	1.385 (6)
O1—C4	1.221 (6)	C7B—H7B1	0.9800
C1—H1C	0.9800	C7B—H7B2	0.9800
C1—H1D	0.9800	C7B—H7B3	0.9800
C1—H1E	0.9800	C8B—C9B	1.528 (6)
C2—C3	1.476 (8)	C8B—C6C	1.541 (6)
C2—C4	1.522 (7)	C8B—H8B	1.0000
C2—H2	1.0000	C9B—C10B	1.525 (7)
C3—H3A	0.9800	C9B—H9B1	0.9900
C3—H3B	0.9800	C9B—H9B2	0.9900
C3—H3C	0.9800	C10B—C11B	1.531 (8)
C4—C5	1.473 (8)	C10B—H10E	0.9900
C5—C10	1.383 (7)	C10B—H10F	0.9900
C5—C6	1.385 (8)	C11B—H11L	0.9800
C6—C7	1.369 (9)	C11B—H11M	0.9800
C6—H6	0.9500	C11B—H11N	0.9800
C7—C8	1.388 (8)	C12B—C17B	1.385 (7)
C7—H7	0.9500	C12B—C13B	1.391 (7)
C8—C9	1.367 (8)	C13B—C14B	1.391 (7)
C8—C11	1.512 (9)	C13B—H13B	0.9500
C9—C10	1.365 (8)	C14B—C15B	1.370 (8)
C9—H9	0.9500	C14B—H14B	0.9500
C10—H10	0.9500	C15B—C16B	1.370 (9)
C11—H11A	0.9800	C15B—H15B	0.9500
C11—H11B	0.9800	C16B—C17B	1.398 (7)
C11—H11C	0.9800	C16B—H16B	0.9500
P1A—O3A	1.462 (3)	C17B—H17B	0.9500
P1A—O1A	1.582 (3)	C1C—C6C	1.393 (6)
P1A—O2A	1.592 (3)	C1C—C2C	1.396 (6)
P1A—C12A	1.774 (4)	C1C—H1C1	0.9500
O1A—C3A	1.422 (5)	C2C—C3C	1.384 (6)
O2A—C5B	1.410 (5)	C2C—C8C	1.529 (6)
P1B—O3B	1.465 (3)	C3C—C4C	1.392 (6)
P1B—O1B	1.582 (3)	C4C—C5C	1.392 (6)
P1B—O2B	1.598 (3)	C4C—C7C	1.506 (6)
P1B—C12B	1.771 (5)	C5C—C6C	1.391 (6)
O1B—C3B	1.409 (5)	C7C—H7C1	0.9800
O2B—C5C	1.415 (5)	C7C—H7C2	0.9800
P1C—O3C	1.465 (3)	C7C—H7C3	0.9800
P1C—O1C	1.584 (3)	C8C—C6D	1.525 (5)
P1C—O2C	1.592 (3)	C8C—C9C	1.528 (6)
P1C—C12C	1.779 (5)	C8C—H8C	1.0000
O1C—C3C	1.419 (5)	C9C—C10C	1.501 (6)
O2C—C5D	1.418 (5)	C9C—H9C1	0.9900
P1D—O3D	1.463 (3)	C9C—H9C2	0.9900
P1D—O1D	1.590 (3)	C10C—C11C	1.533 (7)
P1D—O2D	1.592 (3)	C10C—H10H	0.9900
P1D—C12D	1.781 (4)	C10C—H10G	0.9900
O1D—C3D	1.416 (5)	C11C—H11O	0.9800
O2D—C5A	1.418 (5)	C11C—H11P	0.9800
C10D—C11D	1.515 (7)	C11C—H11Q	0.9800
C10D—C9D	1.534 (6)	C12C—C13C	1.384 (8)
C10D—H10K	0.9900	C12C—C17C	1.389 (7)
C10D—H10J	0.9900	C13C—C14C	1.413 (9)
C2A—C1A	1.393 (6)	C13C—H13C	0.9500
C2A—C3A	1.396 (6)	C14C—C15C	1.352 (9)
C2A—C8A	1.529 (6)	C14C—H14C	0.9500
C3A—C4A	1.375 (6)	C15C—C16C	1.340 (9)
C4A—C5A	1.398 (5)	C15C—H15C	0.9500
C4A—C7A	1.500 (6)	C16C—C17C	1.389 (7)
C5A—C6A	1.394 (5)	C16C—H16C	0.9500
C6A—C1A	1.391 (6)	C17C—H17C	0.9500
C6A—C8D	1.518 (6)	C1D—C2D	1.395 (5)
C7A—H7A1	0.9800	C1D—C6D	1.406 (6)
C7A—H7A2	0.9800	C1D—H1D1	0.9500
C7A—H7A3	0.9800	C2D—C3D	1.392 (6)
C8A—C9E	1.524 (6)	C2D—C8D	1.524 (6)
C8A—C9A	1.524 (6)	C3D—C4D	1.387 (6)
C8A—C6B	1.535 (6)	C4D—C5D	1.391 (6)
C8A—H8A	1.0000	C4D—C7D	1.497 (6)
C9A—C10A	1.413 (10)	C5D—C6D	1.388 (6)
C9A—H9A1	0.9900	C7D—H7D1	0.9800
C9A—H9A2	0.9900	C7D—H7D2	0.9800
C10A—C11A	1.483 (15)	C7D—H7D3	0.9800
C10A—H10A	0.9900	C8D—C9D	1.545 (6)
C10A—H10B	0.9900	C8D—H8D	1.0000
C11A—H11D	0.9800	C9D—H9D1	0.9900
C11A—H11E	0.9800	C9D—H9D2	0.9900
C11A—H11F	0.9800	C1A—H1A1	0.9500
C9E—C10E	1.379 (11)	C11D—H11R	0.9800
C9E—H9E1	0.9900	C11D—H11S	0.9800
C9E—H9E2	0.9900	C11D—H11T	0.9800
C10E—C11E	1.531 (17)	C12D—C17D	1.351 (6)
C10E—H10C	0.9900	C12D—C13D	1.387 (7)
C10E—H10D	0.9900	C13D—C14D	1.351 (8)
C11E—H11G	0.9800	C13D—H13D	0.9500
C11E—H11H	0.9800	C14D—C15D	1.367 (9)
C11E—H11I	0.9800	C14D—H14D	0.9500
C12A—C17A	1.371 (7)	C15D—C16D	1.333 (8)
C12A—C13A	1.376 (7)	C15D—H15D	0.9500
C13A—C14A	1.386 (8)	C16D—C17D	1.389 (7)
C13A—H13A	0.9500	C16D—H16D	0.9500
C14A—C15A	1.369 (9)	C17D—H17D	0.9500
C14A—H14A	0.9500	O1S—C1S	1.449 (5)
C15A—C16A	1.361 (9)	O1S—H1S	0.8400
C15A—H15A	0.9500	C1S—H1S1	0.9800
C16A—C17A	1.387 (7)	C1S—H1S2	0.9800
C16A—H16A	0.9500	C1S—H1S3	0.9800
C17A—H17A	0.9500	O2S—C2S	1.425 (5)
C1B—C6B	1.391 (6)	O2S—H2S	0.8400
C1B—C2B	1.403 (6)	C2S—H2S1	0.9800
C1B—H1B1	0.9500	C2S—H2S2	0.9800
C2B—C3B	1.387 (6)	C2S—H2S3	0.9800
C2B—C8B	1.508 (6)		
			
C1—N1—C2	113.1 (4)	C4B—C7B—H7B2	109.5
C1—N1—H1A	109.0	H7B1—C7B—H7B2	109.5
C2—N1—H1A	109.0	C4B—C7B—H7B3	109.5
C1—N1—H1B	109.0	H7B1—C7B—H7B3	109.5
C2—N1—H1B	109.0	H7B2—C7B—H7B3	109.5
H1A—N1—H1B	107.8	C2B—C8B—C9B	113.9 (4)
N1—C1—H1C	109.5	C2B—C8B—C6C	109.0 (3)
N1—C1—H1D	109.5	C9B—C8B—C6C	112.2 (3)
H1C—C1—H1D	109.5	C2B—C8B—H8B	107.1
N1—C1—H1E	109.5	C9B—C8B—H8B	107.1
H1C—C1—H1E	109.5	C6C—C8B—H8B	107.1
H1D—C1—H1E	109.5	C10B—C9B—C8B	113.9 (4)
C3—C2—N1	111.6 (5)	C10B—C9B—H9B1	108.8
C3—C2—C4	111.3 (5)	C8B—C9B—H9B1	108.8
N1—C2—C4	108.6 (4)	C10B—C9B—H9B2	108.8
C3—C2—H2	108.4	C8B—C9B—H9B2	108.8
N1—C2—H2	108.4	H9B1—C9B—H9B2	107.7
C4—C2—H2	108.4	C9B—C10B—C11B	110.1 (5)
C2—C3—H3A	109.5	C9B—C10B—H10E	109.6
C2—C3—H3B	109.5	C11B—C10B—H10E	109.6
H3A—C3—H3B	109.5	C9B—C10B—H10F	109.6
C2—C3—H3C	109.5	C11B—C10B—H10F	109.6
H3A—C3—H3C	109.5	H10E—C10B—H10F	108.2
H3B—C3—H3C	109.5	C10B—C11B—H11L	109.5
O1—C4—C5	122.5 (5)	C10B—C11B—H11M	109.5
O1—C4—C2	117.7 (5)	H11L—C11B—H11M	109.5
C5—C4—C2	119.7 (4)	C10B—C11B—H11N	109.5
C10—C5—C6	117.9 (5)	H11L—C11B—H11N	109.5
C10—C5—C4	119.5 (5)	H11M—C11B—H11N	109.5
C6—C5—C4	122.6 (5)	C17B—C12B—C13B	119.5 (4)
C7—C6—C5	120.1 (5)	C17B—C12B—P1B	118.3 (4)
C7—C6—H6	120.0	C13B—C12B—P1B	122.2 (4)
C5—C6—H6	120.0	C12B—C13B—C14B	120.1 (5)
C6—C7—C8	121.8 (6)	C12B—C13B—H13B	119.9
C6—C7—H7	119.1	C14B—C13B—H13B	119.9
C8—C7—H7	119.1	C15B—C14B—C13B	119.6 (5)
C9—C8—C7	117.4 (6)	C15B—C14B—H14B	120.2
C9—C8—C11	120.3 (5)	C13B—C14B—H14B	120.2
C7—C8—C11	122.3 (6)	C16B—C15B—C14B	121.3 (5)
C10—C9—C8	121.4 (5)	C16B—C15B—H15B	119.4
C10—C9—H9	119.3	C14B—C15B—H15B	119.4
C8—C9—H9	119.3	C15B—C16B—C17B	119.5 (6)
C9—C10—C5	121.3 (6)	C15B—C16B—H16B	120.2
C9—C10—H10	119.4	C17B—C16B—H16B	120.2
C5—C10—H10	119.4	C12B—C17B—C16B	120.0 (5)
C8—C11—H11A	109.5	C12B—C17B—H17B	120.0
C8—C11—H11B	109.5	C16B—C17B—H17B	120.0
H11A—C11—H11B	109.5	C6C—C1C—C2C	122.0 (4)
C8—C11—H11C	109.5	C6C—C1C—H1C1	119.0
H11A—C11—H11C	109.5	C2C—C1C—H1C1	119.0
H11B—C11—H11C	109.5	C3C—C2C—C1C	117.3 (4)
O3A—P1A—O1A	114.32 (17)	C3C—C2C—C8C	122.3 (4)
O3A—P1A—O2A	112.78 (17)	C1C—C2C—C8C	120.4 (4)
O1A—P1A—O2A	105.75 (17)	C2C—C3C—C4C	123.8 (4)
O3A—P1A—C12A	117.9 (2)	C2C—C3C—O1C	119.3 (4)
O1A—P1A—C12A	102.66 (18)	C4C—C3C—O1C	116.9 (4)
O2A—P1A—C12A	101.89 (18)	C3C—C4C—C5C	116.0 (4)
C3A—O1A—P1A	121.3 (2)	C3C—C4C—C7C	121.9 (4)
C5B—O2A—P1A	120.6 (3)	C5C—C4C—C7C	122.1 (4)
O3B—P1B—O1B	113.97 (18)	C6C—C5C—C4C	123.5 (4)
O3B—P1B—O2B	112.80 (17)	C6C—C5C—O2B	119.1 (4)
O1B—P1B—O2B	105.85 (16)	C4C—C5C—O2B	117.4 (4)
O3B—P1B—C12B	117.0 (2)	C5C—C6C—C1C	117.3 (4)
O1B—P1B—C12B	102.63 (19)	C5C—C6C—C8B	121.9 (4)
O2B—P1B—C12B	103.30 (18)	C1C—C6C—C8B	120.8 (4)
C3B—O1B—P1B	121.6 (3)	C4C—C7C—H7C1	109.5
C5C—O2B—P1B	121.3 (2)	C4C—C7C—H7C2	109.5
O3C—P1C—O1C	113.94 (18)	H7C1—C7C—H7C2	109.5
O3C—P1C—O2C	114.15 (18)	C4C—C7C—H7C3	109.5
O1C—P1C—O2C	105.73 (16)	H7C1—C7C—H7C3	109.5
O3C—P1C—C12C	117.3 (2)	H7C2—C7C—H7C3	109.5
O1C—P1C—C12C	103.55 (19)	C6D—C8C—C9C	114.9 (3)
O2C—P1C—C12C	100.50 (19)	C6D—C8C—C2C	108.1 (3)
C3C—O1C—P1C	121.9 (2)	C9C—C8C—C2C	112.3 (3)
C5D—O2C—P1C	122.6 (3)	C6D—C8C—H8C	107.0
O3D—P1D—O1D	114.22 (17)	C9C—C8C—H8C	107.0
O3D—P1D—O2D	113.11 (17)	C2C—C8C—H8C	107.0
O1D—P1D—O2D	105.32 (15)	C10C—C9C—C8C	114.6 (4)
O3D—P1D—C12D	117.0 (2)	C10C—C9C—H9C1	108.6
O1D—P1D—C12D	102.41 (18)	C8C—C9C—H9C1	108.6
O2D—P1D—C12D	103.36 (17)	C10C—C9C—H9C2	108.6
C3D—O1D—P1D	121.0 (2)	C8C—C9C—H9C2	108.6
C5A—O2D—P1D	120.7 (2)	H9C1—C9C—H9C2	107.6
C11D—C10D—C9D	113.0 (4)	C9C—C10C—C11C	111.9 (4)
C11D—C10D—H10K	109.0	C9C—C10C—H10H	109.2
C9D—C10D—H10K	109.0	C11C—C10C—H10H	109.2
C11D—C10D—H10J	109.0	C9C—C10C—H10G	109.2
C9D—C10D—H10J	109.0	C11C—C10C—H10G	109.2
H10K—C10D—H10J	107.8	H10H—C10C—H10G	107.9
C1A—C2A—C3A	116.9 (4)	C10C—C11C—H11O	109.5
C1A—C2A—C8A	121.2 (4)	C10C—C11C—H11P	109.5
C3A—C2A—C8A	121.8 (4)	H11O—C11C—H11P	109.5
C4A—C3A—C2A	124.4 (4)	C10C—C11C—H11Q	109.5
C4A—C3A—O1A	117.3 (3)	H11O—C11C—H11Q	109.5
C2A—C3A—O1A	118.2 (4)	H11P—C11C—H11Q	109.5
C3A—C4A—C5A	115.6 (4)	C13C—C12C—C17C	119.4 (5)
C3A—C4A—C7A	122.4 (4)	C13C—C12C—P1C	119.6 (4)
C5A—C4A—C7A	122.0 (4)	C17C—C12C—P1C	120.8 (4)
C6A—C5A—C4A	123.7 (4)	C12C—C13C—C14C	118.7 (6)
C6A—C5A—O2D	119.2 (3)	C12C—C13C—H13C	120.7
C4A—C5A—O2D	117.0 (3)	C14C—C13C—H13C	120.7
C1A—C6A—C5A	117.1 (4)	C15C—C14C—C13C	120.4 (6)
C1A—C6A—C8D	121.6 (4)	C15C—C14C—H14C	119.8
C5A—C6A—C8D	121.3 (4)	C13C—C14C—H14C	119.8
C4A—C7A—H7A1	109.5	C16C—C15C—C14C	121.0 (6)
C4A—C7A—H7A2	109.5	C16C—C15C—H15C	119.5
H7A1—C7A—H7A2	109.5	C14C—C15C—H15C	119.5
C4A—C7A—H7A3	109.5	C15C—C16C—C17C	120.6 (6)
H7A1—C7A—H7A3	109.5	C15C—C16C—H16C	119.7
H7A2—C7A—H7A3	109.5	C17C—C16C—H16C	119.7
C9E—C8A—C2A	113.2 (4)	C12C—C17C—C16C	119.8 (6)
C9A—C8A—C2A	113.2 (4)	C12C—C17C—H17C	120.1
C9E—C8A—C6B	113.1 (4)	C16C—C17C—H17C	120.1
C9A—C8A—C6B	113.1 (4)	C2D—C1D—C6D	121.2 (4)
C2A—C8A—C6B	108.0 (3)	C2D—C1D—H1D1	119.4
C9E—C8A—H8A	107.4	C6D—C1D—H1D1	119.4
C2A—C8A—H8A	107.4	C3D—C2D—C1D	117.9 (4)
C6B—C8A—H8A	107.4	C3D—C2D—C8D	120.1 (4)
C10A—C9A—C8A	121.6 (6)	C1D—C2D—C8D	122.0 (4)
C10A—C9A—H9A1	106.9	C4D—C3D—C2D	123.8 (4)
C8A—C9A—H9A1	106.9	C4D—C3D—O1D	116.6 (4)
C10A—C9A—H9A2	106.9	C2D—C3D—O1D	119.5 (3)
C8A—C9A—H9A2	106.9	C3D—C4D—C5D	115.5 (4)
H9A1—C9A—H9A2	106.7	C3D—C4D—C7D	122.6 (4)
C9A—C10A—C11A	116.4 (9)	C5D—C4D—C7D	121.9 (4)
C9A—C10A—H10A	108.2	C6D—C5D—C4D	124.5 (4)
C11A—C10A—H10A	108.2	C6D—C5D—O2C	118.2 (4)
C9A—C10A—H10B	108.2	C4D—C5D—O2C	117.3 (4)
C11A—C10A—H10B	108.2	C5D—C6D—C1D	117.1 (4)
H10A—C10A—H10B	107.3	C5D—C6D—C8C	120.8 (4)
C10A—C11A—H11D	109.5	C1D—C6D—C8C	122.1 (4)
C10A—C11A—H11E	109.5	C4D—C7D—H7D1	109.5
H11D—C11A—H11E	109.5	C4D—C7D—H7D2	109.5
C10A—C11A—H11F	109.5	H7D1—C7D—H7D2	109.5
H11D—C11A—H11F	109.5	C4D—C7D—H7D3	109.5
H11E—C11A—H11F	109.5	H7D1—C7D—H7D3	109.5
C10E—C9E—C8A	114.8 (6)	H7D2—C7D—H7D3	109.5
C10E—C9E—H9E1	108.6	C6A—C8D—C2D	109.1 (3)
C8A—C9E—H9E1	108.6	C6A—C8D—C9D	114.1 (4)
C10E—C9E—H9E2	108.6	C2D—C8D—C9D	113.1 (3)
C8A—C9E—H9E2	108.6	C6A—C8D—H8D	106.7
H9E1—C9E—H9E2	107.5	C2D—C8D—H8D	106.7
C9E—C10E—C11E	110.3 (9)	C9D—C8D—H8D	106.7
C9E—C10E—H10C	109.6	C10D—C9D—C8D	112.4 (4)
C11E—C10E—H10C	109.6	C10D—C9D—H9D1	109.1
C9E—C10E—H10D	109.6	C8D—C9D—H9D1	109.1
C11E—C10E—H10D	109.6	C10D—C9D—H9D2	109.1
H10C—C10E—H10D	108.1	C8D—C9D—H9D2	109.1
C10E—C11E—H11G	109.5	H9D1—C9D—H9D2	107.9
C10E—C11E—H11H	109.5	C6A—C1A—C2A	122.2 (4)
H11G—C11E—H11H	109.5	C6A—C1A—H1A1	118.9
C10E—C11E—H11I	109.5	C2A—C1A—H1A1	118.9
H11G—C11E—H11I	109.5	C10D—C11D—H11R	109.5
H11H—C11E—H11I	109.5	C10D—C11D—H11S	109.5
C17A—C12A—C13A	119.6 (4)	H11R—C11D—H11S	109.5
C17A—C12A—P1A	121.1 (4)	C10D—C11D—H11T	109.5
C13A—C12A—P1A	119.2 (4)	H11R—C11D—H11T	109.5
C12A—C13A—C14A	120.2 (5)	H11S—C11D—H11T	109.5
C12A—C13A—H13A	119.9	C17D—C12D—C13D	117.8 (4)
C14A—C13A—H13A	119.9	C17D—C12D—P1D	123.8 (4)
C15A—C14A—C13A	119.7 (6)	C13D—C12D—P1D	118.3 (4)
C15A—C14A—H14A	120.1	C14D—C13D—C12D	120.1 (5)
C13A—C14A—H14A	120.1	C14D—C13D—H13D	119.9
C16A—C15A—C14A	120.2 (5)	C12D—C13D—H13D	119.9
C16A—C15A—H15A	119.9	C13D—C14D—C15D	121.6 (6)
C14A—C15A—H15A	119.9	C13D—C14D—H14D	119.2
C15A—C16A—C17A	120.3 (6)	C15D—C14D—H14D	119.2
C15A—C16A—H16A	119.9	C16D—C15D—C14D	118.6 (5)
C17A—C16A—H16A	119.9	C16D—C15D—H15D	120.7
C12A—C17A—C16A	120.0 (5)	C14D—C15D—H15D	120.7
C12A—C17A—H17A	120.0	C15D—C16D—C17D	120.6 (5)
C16A—C17A—H17A	120.0	C15D—C16D—H16D	119.7
C6B—C1B—C2B	121.9 (4)	C17D—C16D—H16D	119.7
C6B—C1B—H1B1	119.0	C12D—C17D—C16D	121.0 (5)
C2B—C1B—H1B1	119.0	C12D—C17D—H17D	119.5
C3B—C2B—C1B	117.2 (4)	C16D—C17D—H17D	119.5
C3B—C2B—C8B	120.9 (4)	C1S—O1S—H1S	109.5
C1B—C2B—C8B	121.9 (4)	O1S—C1S—H1S1	109.5
C2B—C3B—C4B	124.1 (4)	O1S—C1S—H1S2	109.5
C2B—C3B—O1B	119.0 (4)	H1S1—C1S—H1S2	109.5
C4B—C3B—O1B	116.9 (4)	O1S—C1S—H1S3	109.5
C3B—C4B—C5B	115.4 (4)	H1S1—C1S—H1S3	109.5
C3B—C4B—C7B	121.8 (4)	H1S2—C1S—H1S3	109.5
C5B—C4B—C7B	122.8 (4)	C2S—O2S—H2S	109.5
C6B—C5B—C4B	124.0 (4)	O2S—C2S—H2S1	109.5
C6B—C5B—O2A	119.2 (4)	O2S—C2S—H2S2	109.5
C4B—C5B—O2A	116.8 (4)	H2S1—C2S—H2S2	109.5
C5B—C6B—C1B	117.4 (4)	O2S—C2S—H2S3	109.5
C5B—C6B—C8A	120.5 (4)	H2S1—C2S—H2S3	109.5
C1B—C6B—C8A	122.1 (4)	H2S2—C2S—H2S3	109.5
C4B—C7B—H7B1	109.5		
			
C1—N1—C2—C3	67.3 (6)	C2B—C8B—C9B—C10B	56.6 (5)
C1—N1—C2—C4	−169.7 (4)	C6C—C8B—C9B—C10B	−178.9 (4)
C3—C2—C4—O1	86.1 (7)	C8B—C9B—C10B—C11B	176.7 (5)
N1—C2—C4—O1	−37.1 (7)	O3B—P1B—C12B—C17B	38.1 (5)
C3—C2—C4—C5	−93.1 (6)	O1B—P1B—C12B—C17B	163.7 (4)
N1—C2—C4—C5	143.7 (5)	O2B—P1B—C12B—C17B	−86.4 (4)
O1—C4—C5—C10	−0.2 (8)	O3B—P1B—C12B—C13B	−139.4 (4)
C2—C4—C5—C10	179.0 (5)	O1B—P1B—C12B—C13B	−13.9 (4)
O1—C4—C5—C6	179.6 (6)	O2B—P1B—C12B—C13B	96.0 (4)
C2—C4—C5—C6	−1.2 (8)	C17B—C12B—C13B—C14B	1.3 (7)
C10—C5—C6—C7	2.1 (9)	P1B—C12B—C13B—C14B	178.8 (4)
C4—C5—C6—C7	−177.6 (6)	C12B—C13B—C14B—C15B	−0.7 (8)
C5—C6—C7—C8	1.1 (11)	C13B—C14B—C15B—C16B	−0.4 (9)
C6—C7—C8—C9	−3.6 (11)	C14B—C15B—C16B—C17B	0.9 (10)
C6—C7—C8—C11	174.1 (7)	C13B—C12B—C17B—C16B	−0.8 (8)
C7—C8—C9—C10	2.9 (10)	P1B—C12B—C17B—C16B	−178.4 (5)
C11—C8—C9—C10	−174.8 (7)	C15B—C16B—C17B—C12B	−0.3 (9)
C8—C9—C10—C5	0.2 (11)	C6C—C1C—C2C—C3C	1.8 (6)
C6—C5—C10—C9	−2.8 (9)	C6C—C1C—C2C—C8C	−179.4 (3)
C4—C5—C10—C9	176.9 (6)	C1C—C2C—C3C—C4C	0.7 (6)
O3A—P1A—O1A—C3A	−38.7 (3)	C8C—C2C—C3C—C4C	−178.0 (4)
O2A—P1A—O1A—C3A	86.0 (3)	C1C—C2C—C3C—O1C	−176.2 (3)
C12A—P1A—O1A—C3A	−167.6 (3)	C8C—C2C—C3C—O1C	5.1 (5)
O3A—P1A—O2A—C5B	38.9 (4)	P1C—O1C—C3C—C2C	−83.3 (4)
O1A—P1A—O2A—C5B	−86.8 (3)	P1C—O1C—C3C—C4C	99.6 (4)
C12A—P1A—O2A—C5B	166.3 (3)	C2C—C3C—C4C—C5C	−2.4 (6)
O3B—P1B—O1B—C3B	−36.1 (4)	O1C—C3C—C4C—C5C	174.6 (3)
O2B—P1B—O1B—C3B	88.4 (3)	C2C—C3C—C4C—C7C	179.0 (4)
C12B—P1B—O1B—C3B	−163.6 (3)	O1C—C3C—C4C—C7C	−4.0 (6)
O3B—P1B—O2B—C5C	38.8 (4)	C3C—C4C—C5C—C6C	1.7 (6)
O1B—P1B—O2B—C5C	−86.5 (3)	C7C—C4C—C5C—C6C	−179.7 (4)
C12B—P1B—O2B—C5C	166.0 (3)	C3C—C4C—C5C—O2B	−176.8 (3)
O3C—P1C—O1C—C3C	−44.2 (4)	C7C—C4C—C5C—O2B	1.8 (6)
O2C—P1C—O1C—C3C	82.0 (3)	P1B—O2B—C5C—C6C	80.1 (4)
C12C—P1C—O1C—C3C	−172.8 (3)	P1B—O2B—C5C—C4C	−101.3 (4)
O3C—P1C—O2C—C5D	41.0 (4)	C4C—C5C—C6C—C1C	0.7 (6)
O1C—P1C—O2C—C5D	−85.0 (3)	O2B—C5C—C6C—C1C	179.1 (3)
C12C—P1C—O2C—C5D	167.5 (3)	C4C—C5C—C6C—C8B	180.0 (4)
O3D—P1D—O1D—C3D	−36.7 (3)	O2B—C5C—C6C—C8B	−1.5 (5)
O2D—P1D—O1D—C3D	88.0 (3)	C2C—C1C—C6C—C5C	−2.5 (6)
C12D—P1D—O1D—C3D	−164.2 (3)	C2C—C1C—C6C—C8B	178.2 (3)
O3D—P1D—O2D—C5A	38.3 (3)	C2B—C8B—C6C—C5C	−89.6 (5)
O1D—P1D—O2D—C5A	−87.1 (3)	C9B—C8B—C6C—C5C	143.3 (4)
C12D—P1D—O2D—C5A	165.8 (3)	C2B—C8B—C6C—C1C	89.7 (4)
C1A—C2A—C3A—C4A	0.2 (6)	C9B—C8B—C6C—C1C	−37.4 (5)
C8A—C2A—C3A—C4A	−178.6 (4)	C3C—C2C—C8C—C6D	88.4 (4)
C1A—C2A—C3A—O1A	−176.6 (3)	C1C—C2C—C8C—C6D	−90.4 (4)
C8A—C2A—C3A—O1A	4.6 (6)	C3C—C2C—C8C—C9C	−143.8 (4)
P1A—O1A—C3A—C4A	99.2 (4)	C1C—C2C—C8C—C9C	37.5 (5)
P1A—O1A—C3A—C2A	−83.8 (4)	C6D—C8C—C9C—C10C	−62.4 (5)
C2A—C3A—C4A—C5A	−1.7 (6)	C2C—C8C—C9C—C10C	173.4 (4)
O1A—C3A—C4A—C5A	175.1 (3)	C8C—C9C—C10C—C11C	−176.4 (4)
C2A—C3A—C4A—C7A	179.5 (4)	O3C—P1C—C12C—C13C	8.6 (5)
O1A—C3A—C4A—C7A	−3.7 (6)	O1C—P1C—C12C—C13C	135.1 (4)
C3A—C4A—C5A—C6A	1.2 (6)	O2C—P1C—C12C—C13C	−115.7 (4)
C7A—C4A—C5A—C6A	−180.0 (4)	O3C—P1C—C12C—C17C	−177.2 (4)
C3A—C4A—C5A—O2D	−177.1 (3)	O1C—P1C—C12C—C17C	−50.8 (4)
C7A—C4A—C5A—O2D	1.7 (5)	O2C—P1C—C12C—C17C	58.4 (4)
P1D—O2D—C5A—C6A	81.8 (4)	C17C—C12C—C13C—C14C	−0.2 (8)
P1D—O2D—C5A—C4A	−99.8 (4)	P1C—C12C—C13C—C14C	174.0 (4)
C4A—C5A—C6A—C1A	0.7 (6)	C12C—C13C—C14C—C15C	0.2 (9)
O2D—C5A—C6A—C1A	179.0 (3)	C13C—C14C—C15C—C16C	0.2 (10)
C4A—C5A—C6A—C8D	179.9 (3)	C14C—C15C—C16C—C17C	−0.6 (9)
O2D—C5A—C6A—C8D	−1.9 (5)	C13C—C12C—C17C—C16C	−0.2 (7)
C1A—C2A—C8A—C9E	35.4 (5)	P1C—C12C—C17C—C16C	−174.3 (4)
C3A—C2A—C8A—C9E	−145.8 (4)	C15C—C16C—C17C—C12C	0.6 (8)
C1A—C2A—C8A—C9A	35.4 (5)	C6D—C1D—C2D—C3D	1.0 (6)
C3A—C2A—C8A—C9A	−145.8 (4)	C6D—C1D—C2D—C8D	179.1 (4)
C1A—C2A—C8A—C6B	−90.7 (4)	C1D—C2D—C3D—C4D	−1.1 (6)
C3A—C2A—C8A—C6B	88.1 (4)	C8D—C2D—C3D—C4D	−179.2 (4)
C2A—C8A—C9A—C10A	172.8 (6)	C1D—C2D—C3D—O1D	−178.0 (3)
C6B—C8A—C9A—C10A	−63.9 (7)	C8D—C2D—C3D—O1D	3.9 (6)
C8A—C9A—C10A—C11A	−175.6 (8)	P1D—O1D—C3D—C4D	99.1 (4)
C2A—C8A—C9E—C10E	70.3 (7)	P1D—O1D—C3D—C2D	−83.9 (4)
C6B—C8A—C9E—C10E	−166.4 (6)	C2D—C3D—C4D—C5D	0.2 (6)
C8A—C9E—C10E—C11E	171.0 (8)	O1D—C3D—C4D—C5D	177.2 (3)
O3A—P1A—C12A—C17A	−155.6 (4)	C2D—C3D—C4D—C7D	−177.7 (4)
O1A—P1A—C12A—C17A	−29.0 (5)	O1D—C3D—C4D—C7D	−0.7 (6)
O2A—P1A—C12A—C17A	80.4 (5)	C3D—C4D—C5D—C6D	0.9 (6)
O3A—P1A—C12A—C13A	28.1 (5)	C7D—C4D—C5D—C6D	178.8 (4)
O1A—P1A—C12A—C13A	154.7 (4)	C3D—C4D—C5D—O2C	−175.3 (3)
O2A—P1A—C12A—C13A	−95.9 (5)	C7D—C4D—C5D—O2C	2.6 (6)
C17A—C12A—C13A—C14A	−2.4 (9)	P1C—O2C—C5D—C6D	87.6 (4)
P1A—C12A—C13A—C14A	173.9 (5)	P1C—O2C—C5D—C4D	−96.0 (4)
C12A—C13A—C14A—C15A	4.8 (11)	C4D—C5D—C6D—C1D	−1.0 (6)
C13A—C14A—C15A—C16A	−4.6 (11)	O2C—C5D—C6D—C1D	175.2 (3)
C14A—C15A—C16A—C17A	2.0 (11)	C4D—C5D—C6D—C8C	176.3 (4)
C13A—C12A—C17A—C16A	−0.2 (9)	O2C—C5D—C6D—C8C	−7.5 (6)
P1A—C12A—C17A—C16A	−176.5 (5)	C2D—C1D—C6D—C5D	0.0 (6)
C15A—C16A—C17A—C12A	0.4 (11)	C2D—C1D—C6D—C8C	−177.3 (4)
C6B—C1B—C2B—C3B	1.7 (6)	C9C—C8C—C6D—C5D	148.0 (4)
C6B—C1B—C2B—C8B	−179.2 (4)	C2C—C8C—C6D—C5D	−85.7 (5)
C1B—C2B—C3B—C4B	−1.9 (6)	C9C—C8C—C6D—C1D	−34.8 (5)
C8B—C2B—C3B—C4B	179.0 (4)	C2C—C8C—C6D—C1D	91.5 (4)
C1B—C2B—C3B—O1B	−179.7 (3)	C1A—C6A—C8D—C2D	88.8 (4)
C8B—C2B—C3B—O1B	1.2 (6)	C5A—C6A—C8D—C2D	−90.4 (4)
P1B—O1B—C3B—C2B	−82.8 (4)	C1A—C6A—C8D—C9D	−38.9 (5)
P1B—O1B—C3B—C4B	99.3 (4)	C5A—C6A—C8D—C9D	142.0 (4)
C2B—C3B—C4B—C5B	1.1 (6)	C3D—C2D—C8D—C6A	88.4 (4)
O1B—C3B—C4B—C5B	178.9 (4)	C1D—C2D—C8D—C6A	−89.6 (4)
C2B—C3B—C4B—C7B	−178.7 (4)	C3D—C2D—C8D—C9D	−143.4 (4)
O1B—C3B—C4B—C7B	−0.9 (6)	C1D—C2D—C8D—C9D	38.6 (5)
C3B—C4B—C5B—C6B	0.1 (6)	C11D—C10D—C9D—C8D	−169.4 (5)
C7B—C4B—C5B—C6B	179.9 (4)	C6A—C8D—C9D—C10D	−57.6 (5)
C3B—C4B—C5B—O2A	−177.8 (3)	C2D—C8D—C9D—C10D	176.8 (4)
C7B—C4B—C5B—O2A	2.0 (6)	C5A—C6A—C1A—C2A	−2.4 (6)
P1A—O2A—C5B—C6B	85.2 (4)	C8D—C6A—C1A—C2A	178.4 (4)
P1A—O2A—C5B—C4B	−96.8 (4)	C3A—C2A—C1A—C6A	2.0 (6)
C4B—C5B—C6B—C1B	−0.3 (6)	C8A—C2A—C1A—C6A	−179.2 (4)
O2A—C5B—C6B—C1B	177.5 (3)	O3D—P1D—C12D—C17D	−151.1 (4)
C4B—C5B—C6B—C8A	177.5 (4)	O1D—P1D—C12D—C17D	−25.4 (5)
O2A—C5B—C6B—C8A	−4.7 (6)	O2D—P1D—C12D—C17D	83.9 (5)
C2B—C1B—C6B—C5B	−0.6 (6)	O3D—P1D—C12D—C13D	25.5 (5)
C2B—C1B—C6B—C8A	−178.4 (4)	O1D—P1D—C12D—C13D	151.2 (5)
C9E—C8A—C6B—C5B	145.5 (4)	O2D—P1D—C12D—C13D	−99.5 (5)
C9A—C8A—C6B—C5B	145.5 (4)	C17D—C12D—C13D—C14D	−0.5 (10)
C2A—C8A—C6B—C5B	−88.4 (5)	P1D—C12D—C13D—C14D	−177.3 (6)
C9E—C8A—C6B—C1B	−36.9 (6)	C12D—C13D—C14D—C15D	−4.0 (13)
C9A—C8A—C6B—C1B	−36.9 (6)	C13D—C14D—C15D—C16D	4.8 (12)
C2A—C8A—C6B—C1B	89.2 (5)	C14D—C15D—C16D—C17D	−1.2 (10)
C3B—C2B—C8B—C9B	−144.5 (4)	C13D—C12D—C17D—C16D	4.0 (9)
C1B—C2B—C8B—C9B	36.4 (5)	P1D—C12D—C17D—C16D	−179.4 (5)
C3B—C2B—C8B—C6C	89.4 (5)	C15D—C16D—C17D—C12D	−3.3 (10)
C1B—C2B—C8B—C6C	−89.7 (5)		

### Hydrogen-bond geometry (Å, º)

C*g*1 and C*g*2 are the centroids of the rings C1*B*–C6*B* and C1*D*–C6*D*, respectively.

**Table:**

*D*—H···*A*	*D*—H	H···*A*	*D*···*A*	*D*—H···*A*
N1—H1*A*···O3*A*	0.91	1.91	2.773 (5)	157
N1—H1*B*···O3*B*	0.91	1.99	2.841 (5)	155
C2—H2···O3*D*	1.00	2.25	3.140 (6)	148
C3—H3*A*···O3*C*	0.98	2.49	3.351 (7)	147
C3—H3*B*···O1*S*	0.98	2.55	3.51 (2)	164
O2*S*—H2*S*···Cl1	0.84	2.28	3.105 (5)	169
C1*A*—H1*A*1···Cl1	0.95	2.91	3.847 (4)	170
C1*B*—H1*B*1···Cl1	0.95	2.93	3.870 (5)	170
C1*C*—H1*C*1···Cl1	0.95	2.95	3.888 (5)	170
C1*D*—H1*D*1···Cl1	0.95	2.85	3.782 (5)	168
C9*A*—H9*A*1···Cl1	0.99	2.76	3.738 (5)	172
C9*B*—H9*B*2···Cl1	0.99	2.88	3.870 (4)	175
C9*C*—H9*C*1···Cl1	0.99	2.71	3.701 (5)	175
C9*D*—H9*D*1···Cl1	0.99	2.85	3.838 (5)	178
C1—H1*D*···C*g*1	0.98	2.83	3.672 (7)	145
C17*D*^i^—H17*D*^i^···O1	0.95	2.56	3.204 (6)	125
C10—H10···O1*D*^i^	0.95	2.69	3.555 (4)	152
C9—H9···C*g*2^i^	0.95	2.69	3.594 (5)	159
C14*B*—H14*B*···Cl1^ii^	0.95	2.89	3.697 (6)	143

Symmetry codes: (i) −*x*+1, *y*+1/2, −*z*+3/2; (ii) −*x*, *y*+1/2, −*z*+3/2.
